# Proximity-enabled covalent binding of IL-2 to IL-2Rα selectively activates regulatory T cells and suppresses autoimmunity

**DOI:** 10.1038/s41392-022-01208-3

**Published:** 2023-01-23

**Authors:** Bo Zhang, Jiaqi Sun, Yeshuang Yuan, Dezhong Ji, Yeting Sun, Yudong Liu, Shengjie Li, Xingxing Zhu, Xunyao Wu, Jin Hu, Qiu Xie, Ling Wu, Lulu Liu, Boyang Cheng, Yuanjie Zhang, Lingjuan Jiang, Lidan Zhao, Fei Yu, Wei Song, Min Wang, Yue Xu, Shiliang Ma, Yunyun Fei, Lihe Zhang, Demin Zhou, Xuan Zhang

**Affiliations:** 1 State Key Laboratory of Complex Severe and Rare Diseases, Peking Union Medical College Hospital; Department of Rheumatology, Beijing Hospital, National Center of Gerontology, Institute of Geriatric Medicine, Chinese Academy of Medical Sciences and Peking Union Medical College, Beijing, 100730 China; 2 State Key Laboratory of Natural and Biomimetic Drugs, School of Pharmaceutical Sciences, Peking University, Beijing, 100191 China; 3 Department of Rheumatology, Beijing Hospital, National Center of Gerontology, Institute of Geriatric Medicine, Clinical Immunology Center, Graduate School of Peking Union Medical College, Chinese Academy of Medical Sciences and Peking Union Medical College, Beijing, 100730 China; 4 Department of Rheumatology and Clinical Immunology, Peking Union Medical College Hospital, Chinese Academy of Medical Sciences and Peking Union Medical College, The Ministry of Education Key Laboratory, Beijing, 100730 China

**Keywords:** Rheumatic diseases, Chemical biology, Molecular engineering

## Abstract

Interleukin-2 (IL-2) is a pleiotropic cytokine that orchestrates bidirectional immune responses via regulatory T cells (Tregs) and effector cells, leading to paradoxical consequences. Here, we report a strategy that exploited genetic code expansion-guided incorporation of the latent bioreactive artificial amino acid fluorosulfate-L-tyrosine (FSY) into IL-2 for proximity-enabled covalent binding to IL-2Rα to selectively promote Treg activation. We found that FSY-bearing IL-2 variants, such as L72-FSY, covalently bound to IL-2Rα via sulfur-fluoride exchange when in proximity, resulting in persistent recycling of IL-2 and selectively promoting the expansion of Tregs but not effector cells. Further assessment of L72-FSY-expanded Tregs demonstrated that L72-FSY maintained Tregs in a central memory phenotype without driving terminal differentiation, as demonstrated by simultaneously attenuated expression of lymphocyte activation gene-3 (LAG-3) and enhanced expression of programmed cell death protein-1 (PD-1). Subcutaneous administration of L72-FSY in murine models of pristane-induced lupus and graft-versus-host disease (GvHD) resulted in enhanced and sustained therapeutic efficacy compared with wild-type IL-2 treatment. The efficacy of L72-FSY was further improved by N-terminal PEGylation, which increased its circulatory retention for preferential and sustained effects. This proximity-enabled covalent binding strategy may accelerate the development of pleiotropic cytokines as a new class of immunomodulatory therapies.

## Introduction

Cell-mediated proinflammatory and anti-inflammatory responses are strictly regulated by cytokines, which can serve as potential therapeutic targets. Signalling ligand–receptor complexes are well known to be internalized after interacting on the cell surface and can be either degraded in lysosomes or recycled to the cell surface.^1^ Receptor-mediated endocytosis can be used to modulate the functions of cytokines when they bind to their receptors.^1,2^ To exert sustained effects, cytokines must be continuously administered at artificially high doses, which may lead to nonselective activation and/or serious side effects. Strategies including modifying the trafficking properties of cytokines to enhance their biological activity while avoiding unwanted nonselectivity and/or side effects are promising.^3–5^

Interleukin-2 (IL-2) is a pleiotropic cytokine that orchestrates immune responses by promoting the differential expansion and activation of regulatory T cells (Tregs) and effector T cells (Teffs) to maintain immune homeostasis.^6–8^ IL-2 signals via a high-affinity (Kd ≈10 pM) trimeric IL-2 receptor (IL-2R) composed of IL-2Rα (CD25), IL-2Rβ (CD122) and common γ_c_ receptor subunits (CD132) in a noncovalent manner or an intermediate-affinity (Kd ≈ 1 nM) dimeric IL-2R composed of IL-2Rβ and γ_c_.^6^ Low-dose IL-2 (LD-IL-2) preferentially activates Tregs that express IL-2Rα and may exert therapeutic effects in a number of inflammatory and autoimmune diseases (ADs),^9–12^ such as systemic lupus erythematosus (SLE),^11^ type I diabetes (T1D)^13,14^ and graft-versus-host disease (GvHD).^12,15^ However, IL-2 can also activate Teffs when the dose is increased to improve unsatisfactory efficacy, which may lead to the exacerbation of ADs.^9^ Engineering IL-2 to target specific cell populations with enhanced in vivo properties has great significance for the improvement of IL-2 based therapy, and numerous approaches have been developed by the rational utilization of the differential expression patterns of IL-2R to functionally activate distinct T cell subsets. For instance, monoclonal antibodies against different regions of IL-2 have been developed to selectively target the binding of IL-2 with different receptor subunits.^16–18^ PEGylation is another potential strategy for selectively modulating the effects of IL-2,^19,20^ and PEGylated IL-2 variants that preferentially bind to trimeric or dimeric IL-2R to skew immune homeostasis in favour of Tregs versus Teff cells are being explored in the treatment of autoimmune disease and cancer. Although how IL-2 works is understood, manipulating IL-2 to be as selective and potent as possible remains a topic of ongoing investigation because improved agents are urgently needed in the clinic.^21^

When engaged with its cognate receptor, the IL-2/receptor complex undergoes rapid endocytosis and subsequent dissociation in endosomes. IL-2Rβ and γ_c_ are routed to lysosomes for degradation,^22–24^ while IL-2Rα remains in early endosomes and can then be recycled back to the cell surface.^24^ The trafficking of IL-2 is driven by its relative binding affinity for receptor subunits, providing a key target for enhancing the effect of IL-2 on Tregs. IL-2Rα, however, is not thought to facilitate recycling and sustained signalling due to its low affinity for IL-2 (KD ~ 10 nM), which results in depletion of IL-2 and reduced its therapeutic effectiveness at pharmacokinetic (PK) and therapeutic low-dose levels.^6,23,24^

A few studies have endeavoured to examine recycling and retention to augment IL-2 signalling.^25–31^ A series of high-affinity IL-2Rα-binding IL-2 mutants were developed to enhance biological potency compared with that of wild-type IL-2 (WT-IL-2).^27,28^ In addition, an internalization-impaired IL-2 mutant was identified, which exhibited a prolonged capacity to maintain T-cell proliferation compared to WT-IL-2 in a fashion that was independent of the binding affinity.^26^ Moreover, antagonists of IL-2 and IL-15 with augmented IL-2Rβ binding affinity were generated by attenuating their interaction with γ_c_; this mutation induced defective IL-2Rβ-γ_c_ heterodimerization and theoretically reduced internalization, resulting in inhibition of the binding of endogenous IL-2.^31^ Our group recently demonstrated that the PK and Treg selectivity of IL-2 were significantly improved by site-specific modification of its binding sites, which may have been partly due to the effects of the alterations on internalization and recycling processes.^19^ Given that the effects of these IL-2 mutants are dependent on the binding affinity for IL-2Rα and that marked augmentation is generally required,^28^ such binding still remains noncovalent and reversible in nature. We reasoned that development of an IL-2 variant with the capacity to covalently bind to IL-2Rα with infinite affinity will break the natural limit of IL-2 on the reversible binding with its receptor and may achieve remarkably and unexpected results.

The sulfur-fluoride exchange (SuFEx) is a wonderful reaction for click chemistry with the high positional specificity and physiological reaction conditions.^32^ In this study, we used genetic codon expansion technology to incorporate the latent bioreactive artificial amino acid fluorosulfate-L-tyrosine (FSY)^33,34^ into human IL-2 at desired positions to generate an IL-2 variant with the capacity to covalently bind to IL-2Rα with irreversible and infinite affinity upon assembly. The FSY residue remains inert until it is sufficiently close to the natural target residue, where FSY-bearing IL-2 interacts with its receptor by forming a covalent bond through SuFEx.^32,34^ The covalent IL-2 mutant, exemplified by one candidate with an FSY substitution at the L72 site of IL-2 (L72-FSY), activated Tregs in a preferential and sustained manner. Strikingly, this IL-2 mutant exhibited distinct effects on the phenotype of expanded Tregs and had better efficacy than its WT counterpart in ameliorating disease severity in murine disease models of SLE and GvHD. In addition, we demonstrated that the therapeutic effect of L72-FSY was further improved by N-terminal PEGylation, which enhanced its circulatory persistence. This study is the first to apply a covalent binding strategy to modify the trafficking of a cytokine to generate selective agonists, and the resultant agonist showed strikingly high binding selectivity and treatment efficacy for ADs. Our work opens a new direction for engineering cytokines to significantly improve their agonistic or antagonistic effects via a covalent binding strategy.

## Results

### Site-specific incorporation of FSY into IL-2

**Fig. 1:**
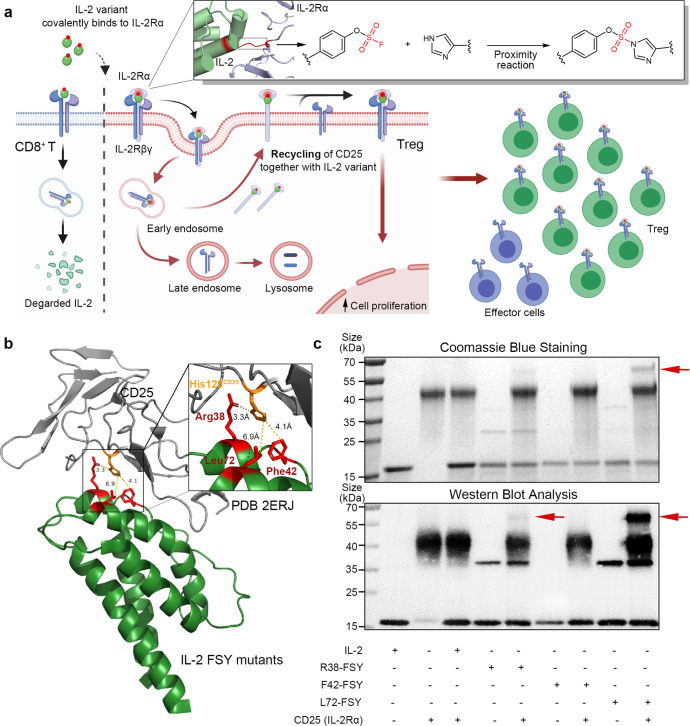
Genetic code expansion-mediated incorporation of FSY to generate an IL-2 variant that covalently binds to IL-2Rα. **a** Schematic representation of the selective covalent binding-mediated augmentation of the recycling and extracellular maintenance of IL-2 on Tregs. Upon the binding of IL-2 to its high-affinity trimeric IL-2 receptor expressed on Tregs, the quaternary IL-2−receptor complex undergoes endocytosis and subsequent dissociation in the early endosome. IL-2Rα can then be recycled back to the cell surface, whereas IL-2 associated with other subunits undergoes degradation in the lysosome. The incorporation of FSY into IL-2 allows it to covalently bind to IL-2α with infinite affinity, thereby distinctly augmenting the recycling of IL-2 and promoting IL-2 signalling sustainably preferentially on Tregs. **b** Crystal structure of human IL-2 assembled together with IL-2α (PDB number: 2ERJ) showing selected sites in stick form. All sites were within 10 Å of the selected H120 site of IL-2Rα. **c** Analysis of the covalent binding of FSY-bearing IL-2 variants to IL-2α. Equal doses of purified WT-IL-2 and IL-2 variants were incubated with the His-tagged ectodomain of IL-2Rα (IL-2Rα-His) at room temperature overnight, and the denatured samples were then analysed by Coomassie blue and western blotting (WB). The anti-His antibody was used as the primary WB antibody. The red arrows indicate the covalent crosslinking of IL-2 and IL-2Rα. See also Supplementary Fig. 2

To skew the trafficking fate of IL-2 towards recycling and prolong its dwell time on the surface of Tregs, we aimed to engineer an IL-2 variant with the capacity to selectively and covalently bind to IL-2Rα upon engagement. We reasoned that the incorporation of the artificial amino acid FSY into IL-2 at or near its binding interface with IL-2Rα would enable the selective reactivity of FSY with a natural residue in close proximity via SuFEx, thus allowing IL-2 to covalently bind to IL-2Rα in a stable manner and substantially augment the recycling of IL-2 (Fig. 1a). Based on the crystal structure of the IL-2/IL-2R complex,^35,36^ three constitutive residues on IL-2 (R38, F42 and L72, all within 10 Å of the key histidine 120 (H120) residue in IL-2Rα) were selected for FSY incorporation (Fig. 1b). In brief, we mutated a triplet codon within the human IL-2 gene to an amber stop codon (UAG), and the resultant plasmid was then cotransformed into an OrigamiB (DE3) strain with a helper plasmid containing an orthogonal amber tRNA^Pyl^/RS^FSY^ pair for site-specific incorporation of the artificial amino acid FSY into IL-2^37,38^ (Supplementary Fig. 1a). The FSY-bearing IL-2 mutants were purified by affinity chromatography, yielding approximately 1 to 3 mg/L per mutant depending on the incorporation site of FSY. The site-specific incorporation of FSY into IL-2 was validated by mass spectrometry (MS) (Supplementary Fig. 1b).

### FSY-bearing IL-2 mutants covalently bind to IL-2Rα in a selective manner in vitro

We first determined whether the FSY-incorporated IL-2 mutants could covalently bind to human IL-2Rα in vitro. Equal amounts of the IL-2 mutants were incubated with the ectodomain of IL-2Rα (1 mg/ml) at room temperature overnight, and WT-IL-2 was used as a negative control (Fig. 1c). Both IL-2 mutants and the ectodomain of IL-2Rα were appended with a 6 × histidine (His) tag at their C-terminus for detection with an anti-His-tag antibody. The crosslinking of IL-2 with the ectodomain of IL-2Rα was identified based on the appearance of an ~60 kDa band, which corresponded to the sum of IL-2 (~15 kDa) plus the ectodomain of his-tagged IL-2Rα (~45 kDa). Incubation of R38-FSY-IL-2 (R38-FSY) and L72-FSY-IL-2 (L72-FSY) with the ectodomain of IL-2Rα resulted in the formation of covalent complexes, as determined by Coomassie blue staining and western blot analysis under denaturing conditions (Fig. 1c). Notably, L72-FSY displayed better covalent crosslinking efficiency than R38-FSY. In contrast, no such product was detected for either WT-IL-2 or F42-FSY, suggesting that FSY reacted with IL-2Rα in a proximity-based manner. To verify whether IL-2(FSY) covalently targeted the proximal H120 of IL-2Rα as designed, we mutated H120 of IL-2Rα to Ala and found that the resultant IL-2Rα (H120A) with a human IgG1-Fc tag did not form a covalent complex with L72-FSY (Supplementary Fig. 2a). The chemical crosslinking between L72-FSY and IL-2Rα was further validated by tandem MS, in which the representative cross-linked sequence of the peptide containing both FSY of IL-2 and H120 of IL-2Rα was detected (Supplementary Fig. 2b).

We then sought to determine whether the FSY-bearing IL-2 mutant could covalently bind to IL-2Rα at the cellular level using engineered IL-2Rα-expressing YT cells (CD25-YT) as previously described.^39^ L72-FSY was used in subsequent experiments due to its higher covalent crosslinking efficiency relative to R38-FSY. L72-FSY was incubated with CD25-YT cells at the indicated times and concentrations, followed by western blot analysis using an anti-IL-2Rα antibody. As expected, L72-FSY covalently crosslinked to the engineered full-length IL-2Rα expressed on the surface of CD25-YT cells, whereas no crosslinked product was observed in WT-IL-2-treated cells (Supplementary Fig. 3a). Further covalent crosslinking characterization demonstrated that the crosslinking efficiency increased as the L72-FSY concentration increased (0.0032 to 10 μg/ml, 5-fold dilution) and generally occurred within 1 to 4 h under physiological conditions (2 μg/ml, 37 °C, PBS, pH = 7.4), as determined by cellular and molecular analyses (Supplementary Fig. 3a, b). In addition, the crosslinking efficiency was strictly dependent on the reaction temperature, as no obvious crosslinking band was observed when proteins of L72-FSY and IL-2Rα-His were incubated at 4 °C, even when the reaction period was extended overnight (more than 16 h, Supplementary Fig. 3c).

Taken together, these results suggest that site-specific incorporation of FSY into IL-2, particularly L72-FSY, enables the IL-2 mutant to covalently bind to IL-2Rα via the H120 residue. Covalent binding was achieved efficiently and specifically under physiological conditions.

### FSY-mediated covalent binding increased the recycling and cell surface dwell time of IL-2 on Tregs

**Fig. 2:**
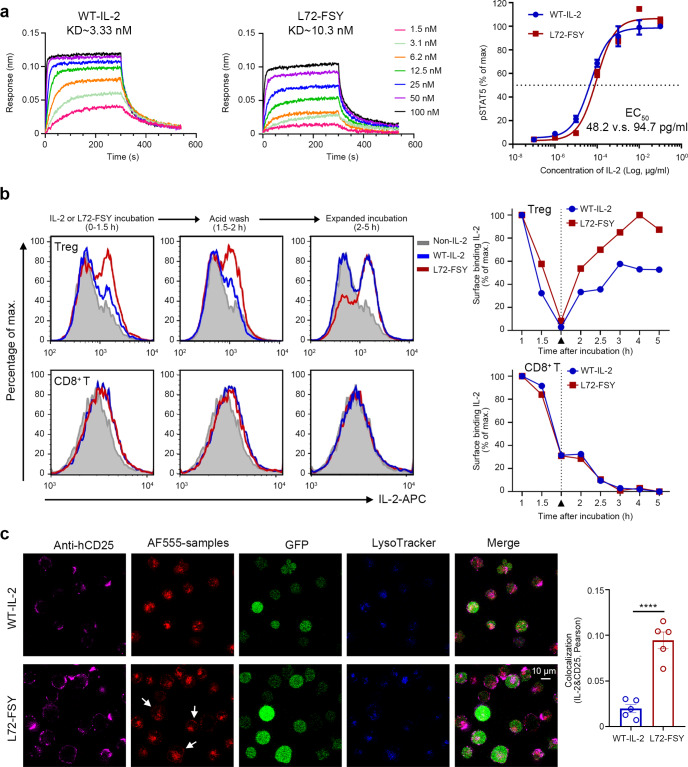
FSY-mediated covalent binding specifically augmented the recycling of IL-2 on Treg cells. **a** Characterization of the effects of FSY mutation on the bioactivity of IL-2. The binding affinities of L72-FSY versus WT-IL-2 were evaluated by biolayer interferometry with biosensors bearing extracellular domains of IL-2Rα. Dose-dependent assessment of the abilities of L72-FSY and WT-IL-2 to activate YT cells expressing CD25-eGFP (CD25-YT) were determined by the pSTAT5 assay. The kinetic parameters and 50% effective concentrations (EC50) of CD25-YT cells in response to each sample were calculated. See also Supplementary Table 1. **b** Flow cytometry assessment of surface IL-2 on Tregs and CD8^+^ T cells in response to L72-FSY versus WT-IL-2. The isolated Tregs or CD8^+^ T cells were incubated with saturating amounts of the samples for 1.5 h. The cells were then washed with acid buffer and replated without cytokines. Tregs were isolated and expanded ex vivo for 9 days prior to the assay. The cells treated at the indicated time points were washed, stained with an anti-IL-2-APC antibody, and analysed by flow cytometry. The cells treated with PBS (non-IL-2) were used as negative control for all samples. The results from one representative donor of three are shown. **c** Characterization of the effects of IL-2 upon covalent binding to the trimeric IL-2R receptor. CD25-YT (green) were incubated at 37 °C in the presence of saturating Alexa Fluor 555-labelled L72-FSY or WT-IL-2 (red) for 6 h. The acidic compartments were stained with LysoTracker blue BND-22 (blue), and surface IL-2Rα expression was detected with an anti-IL-2α antibody followed by a FITC-conjugated secondary antibody (pink). The discrete punctate structures indicated by the white arrow indicated that surface IL-2 colocalized with IL-2Rα. The colocalization of IL-2Rα and IL-2 was determined by Pearson coefficient analysis, and the data are presented as the mean ± SEM derived from five randomly selected cell regions (*n* = 5). The *p* values were determined by two-tailed unpaired Student’s t tests, ****p ≤ 0.0001. Representative results from one of two experiments are shown

We next assessed whether the covalent binding of L72-FSY could prolong the dwell time of IL-2 on the surface of Tregs and sustainably augment downstream signals. We first determined the effects of the L72-FSY mutant on the bioactivity of IL-2 using an affinity binding assay performed with biolayer interferometry (BLI). The affinity of L72-FSY for IL-2Rα was mildly decreased (approximately 3-fold) compared with that of WT-IL-2 (Fig. 2a), which was consistent with previous studies that reported that L72 of IL-2 plays a role in receptor binding.^35,36^ Importantly, FSY incorporation did not significantly compromise the bioactivity of IL-2, as determined by STAT5 phosphorylation (pSTAT5) in CD25-YT cells in response to titrated WT-IL-2 or L72-FSY (Fig. 2a and Supplementary Table 1). To explore the trafficking of L72-FSY upon binding with IL-2Rα, a pulse assay was utilized to mimic transient cytokine exposure upon bolus administration.^24^ Briefly, Tregs (CD3^+^CD4^+^CD8^-^CD25^high^CD127^low^) or CD8^+^ T cells (CD3^+^CD4^-^CD8^+^) were isolated (Supplementary Fig. 4a) and cultured with saturating doses of cytokines for 1.5 h, followed by acid washing and replating without cytokines. As expected, Tregs displayed higher levels of IL-2Rα than CD8^+^ T cells (Supplementary Fig. 4b). Upon extended culture at 37 °C, the substantial reappearance of L72-FSY but not WT-IL-2 on the surface of Tregs was observed. This phenotype was dependent on the amount of available IL-2Rα, as the reappearance of neither L72-FSY nor WT-IL-2 was observed on the surface of CD8^+^ T cells over an extended incubation period (Fig. 2b). The marked improvement in the persistence of surface IL-2 was further verified by confocal microscopy, which revealed discrete punctate structures that colocalized with IL-2Rα when CD25-YT cells were incubated with L72-FSY but not WT-IL-2 for 6 h at 37 °C (Fig. 2c). These results collectively suggest that FSY-mediated covalent binding to IL-2Rα, particularly resulting in L72-FSY, indeed endows IL-2 with a sustainable ability to prolong the recycling and cell surface dwell time on Tregs.

### L72-FSY preferentially promoted the proliferation of Tregs but not Teffs in vitro and in vivo

**Fig. 3:**
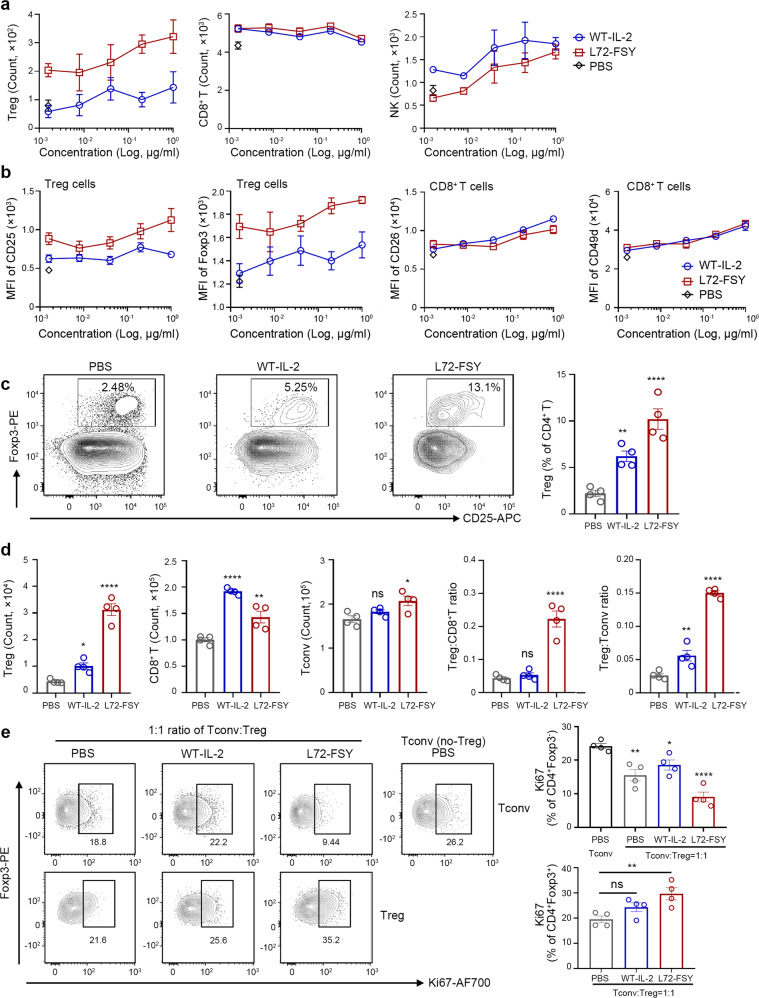
L72-FSY preferentially promotes Tregs in vitro and in vivo. (**a**, **b)** L72-FSY preferentially promoted the proliferation and activation of Tregs over CD8^+^ T cells in vitro. Human PBMCs (hPBMCs) were incubated with samples at the indicated concentrations for 6 h, and the cells were then washed with acid buffer and cultured without cytokines for three days. The symbol is plotted as the mean of triplicate wells, with the error bars representing the SEM (*n* = 3), and one representative donor of three is shown. See also Supplementary Fig. 5. **a** Dose-dependent changes in the numbers of Tregs, CD8^+^ T cells and NK cells following incubation with hPBMCs in the presence of the indicated samples. **b** The expression of IL-2-inducible proteins on Tregs (CD25 and Foxp3) and CD8^+^ T cells (CD26 and CD49b) in the presence of the indicated concentrations of the indicated samples was evaluated. **(c**, **d)** L72-FSY preferentially promoted the proliferation and activation of Tregs in a humanized NSG model in vivo. NSG mice were injected with hPBMCs, followed by subcutaneous administration of 2 μg of WT-IL-2 or L72-FSY daily for ten consecutive days. Splenocytes were harvested five days after the last injection for flow cytometry analysis. See also Supplementary Fig. 7a-b. **c** Representative flow plots and a bar graph reflecting the changes in the percentage of Tregs among total CD4^+^ T cells after the indicated treatments. **d** Bar graphs showing the numbers of Tregs, CD8^+^ T cells, and Tconv cells and the ratios of Tregs:Tconv cells and Tregs:CD8^+^ T cells in the spleens of mice receiving the indicated treatments. **e** L72-FSY selectively potentiated the growth of adoptively transferred Tregs and suppressed transferred Tconv cells in the recipient mouse. NSG mice were cotransferred with isolated human CD4^+^ Tconv and Treg cells at a ratio of 1:1, followed by the administration of 2 μg of WT-IL-2 or L72-FSY daily for five consecutive days. The Tregs were expanded for two weeks after isolation and labelled with carboxyfluorescein succinimidyl ester (CFSE) prior to injection. Representative flow plots and bar graphs reflecting Ki-67^+^ proliferating cells in transferred Tregs and Tconv cells after the indicated treatment. See also Supplementary Fig. 7c. Data are representative of 4 (panel **c** **d**) or 2 (panel **e**) independent experiments and are presented as the mean ± SEM of four mice per group (*n* = 4). The *p* values were determined by one-way ANOVA (Dunnett’s multiple-comparison test compared with the PBS control), *p ≤ 0.05, **p ≤ 0.01, ****p ≤ 0.0001

To assess whether covalent IL-2, exemplified by L72-FSY, was capable of preferentially activating Tregs but not Teffs in vitro, we cultured bulk human peripheral blood mononuclear cells (hPBMCs) with serial dilutions of WT-IL-2 or L72-FSY for 6 h and then without cytokines for an additional 72 h. The gating strategies that were used for this assessment, including those for Tregs (CD3^+^CD4^+^CD25^high^Foxp3^+^), CD8^+^ T cells (CD3^+^CD4^-^CD8^+^) and natural killer (NK, CD3^-^CD16^+^56^+^) cells, are provided in Supplementary Fig. 5a. We found that the proportions and numbers of Tregs but not CD8^+^ T or NK cells were strikingly increased in the L72-FSY-treated group (Fig. 3a and Supplementary Fig. 5b). Consistent with this, L72-FSY treatment significantly upregulated the activation markers CD25 and Foxp3 on Tregs but not CD26, CD49d or CD69 on CD8^+^ T and NK cells (Fig. 3b and Supplementary Fig. 5c). The expression levels of CD26, CD49d and CD69 are directly influenced by IL-2 and complemented by CD25 in the assessment of CD8^+^ T cells.^40^ This finding correlated well with the notable and sustained induction of pSTAT5 signalling in Tregs but not in CD8^+^ T cells subjected to pulsed incubation with L72-FSY compared with that of WT-IL-2 (Supplementary Fig. 6). Taken together, these data suggest that L72-FSY preferentially promotes Tregs over effector cells in vitro and that expanded Tregs exhibit increased expression of functional molecular determinants.

We next investigated the effects of L72-FSY in vivo. A humanized mouse model was established by intravenous administration of hPBMCs into immunodeficient NOD-SCID IL-2 receptor gamma null (NSG) mice, as previously described.^17^ The humanized mice were subcutaneously administered 2 μg of WT-IL-2 or L72-FSY daily for ten consecutive days (a total of 10 times) (Supplementary Fig. 7a). Mobilization analysis of splenocytes harvested five days after the last injection revealed that L72-FSY treatment significantly increased the number of Tregs compared to WT-IL-2 treatment, whereas no significant increase in CD8^+^ T or conventional T cells (Tconv cells, defined as CD4^+^ T cells excluding Tregs) was observed. Thus, L72-FSY treatment led to a significant skewing of Tregs over CD8^+^ Teffs and CD4^+^ Tconv cells in the spleen (Fig. 3c, d). The selective expansion of Tregs was accompanied by upregulated expression of activated biomarkers in Tregs but not CD8^+^ T cells in vivo (Supplementary Fig. 7b). To more specifically verify the direct effect of L72-FSY on Treg expansion, we employed a T-cell transfer model in which isolated human CD4^+^ Tconv (CD3^+^CD4^+^CD25^-^) cells and Tregs (CD3^+^CD4^+^CD25^high^CD127^low^) were cotransferred into NSG mice at a 1:1 ratio, followed by administration of either WT-IL-2 or L72-FSY. Mice transferred with Tconv cells with or without Tregs and treated with PBS were used as controls. The Tregs were expanded for two weeks following isolation and labelled with CFSE prior to injection. Consistent with the above results, L72-FSY treatment selectively promoted Treg proliferation, as Tregs but not Tconv cells showed increased Ki-67 expression in L72-FSY-treated mice compared with PBS-treated mice (Fig. 3e). This effect was superior to that of WT-IL-2, as WT-IL-2 treatment induced the proliferation of both Tregs and Tconv cells, which compromised the suppressive activities of Tregs against Tconv cells. This result correlated with the significantly increased Treg division seen in the L72-FSY treatment group compared with the PBS or WT-IL-2 treatment group (Supplementary Fig. 7c). These data suggested that FSY-mediated covalent binding of IL-2 preferentially promotes the activation of Tregs by providing Tregs with homeostatic signals to initiate proliferation without affecting other effector cells.

Because human IL-2 is known to cross-react with murine IL-2Rα (mIL-2Rα),^41^ we next evaluated the covalent targeting specificity of human FSY-bearing IL-2 towards other binding proteins. No covalent crosslinking of FSY-hIL-2 with mIL-2Rα was detected by SDS–PAGE analysis (Supplementary Fig. 8a). Concomitant with this result, a mobilization analysis of the spleens of and blood from treated healthy C57BL/6 mice revealed that the effects of L72-FSY on murine lymphocytes were comparable to those of WT-IL-2 (Supplementary Fig. 8b). L72-FSY even induced a lower level of Tregs, although the difference was not significant, than WT-IL-2, probably due to the mutation of L72 and reduced affinity for murine IL-2α. Additionally, we screened a series of potential sites within murine IL-2 for the incorporation of FSY based on the homologous alignment of the human and murine IL-2 sequences. None of these variants were able to covalently bind to mIL-2Rα (Supplementary Fig. 8c). Collectively, these results suggest that FSY-72 preferentially promotes the activation and proliferation of Tregs but not effector cells in vitro and in vivo in a highly specific, persistent and steady manner.

### Immune characterization of L72-FSY by mass cytometry profiling

To exclude any potential confounding factors due to immunodeficiency in the NSG model, we next evaluated the effects of L72-FSY using an IL-2Rα-humanized B6 mouse model (B-hIL2RA mice), in which the ectodomain of IL-2Rα was completely replaced with humanized IL-2Rα. Time of flight (CyTOF) mass cytometry was utilized to map the immune compartments of the splenocytes. A similar dosing scheme to that in NSG mice was applied, and splenocytes were harvested five days after the last injection. Given that T cells are the major target of IL-2 therapies, we first used T-cell surface markers to analyse the functions and phenotypes of total T cells. Compared to WT-IL-2 treatment, L72-FSY treatment significantly increased the number of Tregs but not other clusters, including CD4^+^ Teffs, CD8^+^ Teffs, γδ-T cells, and double-negative T (DN-T) cells (Supplementary Fig. 9a). Consistently, L72-FSY treatment upregulated functional and proliferative biomarkers, including CD25, Foxp3 and Ki67, on Tregs but not CD8^+^ T cells (Supplementary Fig. 9b). These results were consistent with those of a parallel flow cytometry assay in which L72-FSY treatment significantly increased the number and proportion of Tregs while reducing the proportions of (T-helper 1) Th1 and IL-17-producing T helper (Th17) cells but had minimal effects on the numbers of CD8^+^ T cells, Tconv cells, and natural killer (NK) cells in the spleen of B-hIL2RA mice (Supplementary Fig. 10a). These selective effects resulted in marked skewing of Tregs over CD8^+^ T cells, Tconv cells, and NK cells (Supplementary Fig. 10b). Notably, L72-FSY treatment resulted in persistent selective Treg activation for up to 15 days, which was considerably longer than that in response to WT-IL-2 treatment (Supplementary Fig. 11).

**Fig. 4:**
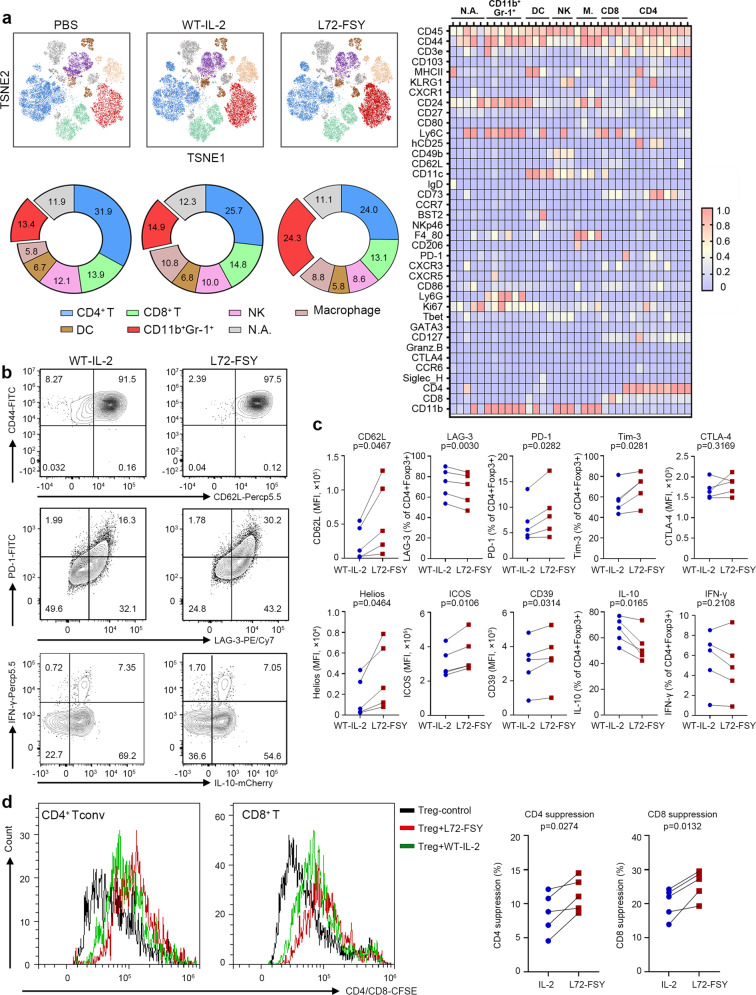
Characterization of L72-FSY versus WT-IL-2 in vivo and in vitro. **a** Profiling of immunocyte populations in the spleen of L72-FSY-treated B-hIL2RA mice by mass cytometry. IL-2Rα-humanized B6 mice (B-hIL2RA mice, three mice per group, *n* = 3) were subcutaneously injected with PBS, WT-IL-2 or L72-FSY (2 μg) daily for ten consecutive days, and splenocytes were harvested five days after the last injection for mass cytometry. Mice treated with PBS were used as a control. The heatmap of FlowSOM-generated splenic immune cell populations excluding B cells (CD45^+^CD19^-^) shows the differential expression of 38 immune markers in the 35 cell clusters. Certain clusters were identified as known cell types within the six cellular clusters according to typically expressed markers. tSNE plots and frequency distributions of immunocytes showing the distinct immune landscape of splenocytes in response to the indicated treatment are depicted. See also Supplementary Fig. 12 and Supplementary Table 2. **b**, **c** Profiling of L72-FSY versus WT-IL-2 on Tregs in vitro. Human Tregs were isolated using fluorescence-activated cell sorting (CD3^+^CD4^+^CD25^high^CD127^low^) and expanded with anti-CD3/CD28 beads in the presence of either L72-FSY or WT-IL-2 (1 μg/ml) ex vivo for two weeks. The samples were re-supplemented every 48 hours. Cells were rested for two days prior to analysis. Representative flow plots (**b**) and plots graph (**c**) showing the indicated biomarker after the indicated treatment. **d** In vitro T cell suppression assay. CFSE-labelled responder CD3^+^ T cells were prestimulated for 1 day with anti-CD3/CD28 beads and IL-2 and cocultured with or without L72-FSY- or WT-IL-2-expanded Tregs (ratio of 2 CD3^+^ T cells to 1 Treg). After coculture for three days, the proliferation of responder CD4^+^ and CD8^+^ T cells was determined by flow cytometry. Representative flow histograms and averaged data are shown. Percent suppression was calculated as the reduction of the division index in the responder-only control. For panel **b**, **c** and **d**, five different donors are represented (*n* = 5), and the *p* values shown were determined by two-tailed paired Student’s t tests

To comprehensively profile the in vivo effect of L72-FSY, splenocytes were harvested five days after different IL-2 treatments and stained with 38 antibodies conjugated to unique metal isotopes to identify all of the major immune cell populations by hierarchical clustering using the FlowSOM algorithm (Supplementary Table 2). Based on the normalized median marker expression in each cluster, a total of 35 clusters belonging to six major cell populations, CD4^+^ T cells, CD8^+^ T cells, macrophages, NK cells, dendritic cells (DCs) and CD11b^+^Gr-1^+^ cells, were identified. Two-dimensional t-stochastic neighbour embedding (tSNE) projections along with heatmaps were used to profile the landscape of each sample (Fig. 4a). In general, WT-IL-2- and FSY-72-treated samples showed significantly higher frequencies of CD8^+^ T cells, NK cells and macrophages than PBS-treated samples. The frequencies of DCs and CD4^+^ T cells were comparable between the WT-IL-2- and FSY-72-treated groups. Intriguingly, we found that the L72-FSY treatment group showed a markedly increased frequency of CD11b^+^Gr-1^+^ cells, a heterogeneous cell subpopulation possibly referred to as myeloid-derived suppressor cells (MDSCs) that are associated with immunosuppression,^42,43^ compared with the WT-IL-2 treatment group (Fig. 4b and Supplementary Fig. S12). This finding was further investigated in parallel by an independent flow cytometry experiment, in which the same dosing scheme as that used in the CyTOF experiment was applied. We first evaluated the suppressive function of CD11b^+^Gr-1^+^ cells isolated from IL-2- or L72-FSY-treated B-hIL2RA mice and found that these cells have the capacity to inhibit the production of IFN-γ by CD4^+^ T cells (Supplementary Fig. 13a). These cells were thus referred to as MDSCs in the following experiment and analysed as previously reported (Supplementary Fig. 13b).^42^ Consistent with the results from CyTOF, we found that L72-FSY treatment significantly increased the proportions and numbers of both polymorphonuclear MDSCs (PMN-MDSCs, CD11b^+^Ly6G^+^Ly6C^low^) and monocytic MDSCs (M-MDSCs, CD11b^+^Ly6G^-^Ly6C^high^) in the spleens of B-hIL2RA mice compared to WT-IL-2 treatment (Supplementary Fig. 13c). The selective promotion of MDSCs by L72-FSY was likely not due to covalent binding to MDSCs, as IL-2Rα is absent in all MDSCs (Supplementary Fig. 13d); therefore, other indirect effects may occur.

### In vitro L72-FSY-expanded Tregs exhibited a central memory phenotype with reduced LAG-3 and elevated PD-1 expression

To further examine the functionality of the expanded Tregs in response to L72-FSY, we examined the phenotype of L72-FSY-expanded Tregs after two weeks of culture in vitro. We found that L72-FSY increased the proportion of Tregs with a central memory phenotype (CD44^high^CD62L^high^) compared with WT-IL-2, as demonstrated by the considerably increased expression of CD62L upon long-term culture (Fig. 4b, c).^44,45^ This result correlated with notably lower induction of lymphocyte activation gene-3 (LAG-3), a marker associated with Treg exhaustion,^46,47^ and higher induction of programmed cell death protein-1 (PD-1), most evidently in central-memory Tregs,^45^ in L72-FSY-expanded Tregs than in their WT-IL-2-expanded counterparts. Moreover, in contrast to WT-IL-2, L72-FSY induced the expression of T-cell immunoglobulin and mucin domain-3 (Tim-3), whereas the expression of cytotoxic T lymphocyte-associated antigen-4 (CTLA-4), another inhibitory molecule, remained stable (Fig. 4b, c). Consistent with the assessment of Foxp3 and CD25, we also found that L72-FSY-expanded Tregs expressed higher levels of stability-related and functional molecules, including Helios, inducible T-cell costimulator (ICOS) and the ectonucleotidase CD39, than WT-IL-2-expanded Tregs and produced less intracellular IL-10, an immunosuppressive cytokine associated with T-cell exhaustion, but similar levels of interferon-γ (IFN-γ) (Fig. 4b, c). Further assessment of suppressive activity demonstrated that L72-FSY-expanded Tregs suppressed the proliferation of both responder CD8^+^ T cells and CD4^+^ T cells, and this suppressive activity was superior to that of control IL-2-expanded Tregs (Fig. 4d). These results collectively provide mechanistic evidence that L72-FSY not only promotes the expansion of Tregs but also maintain Tregs in a central memory phenotype with suppressive function without driving terminal differentiation.

### N-terminal PEGylation prolonged the half-life of L72-FSY and enhanced Treg-selective activation in vivo without compromising covalent binding activity

**Fig. 5:**
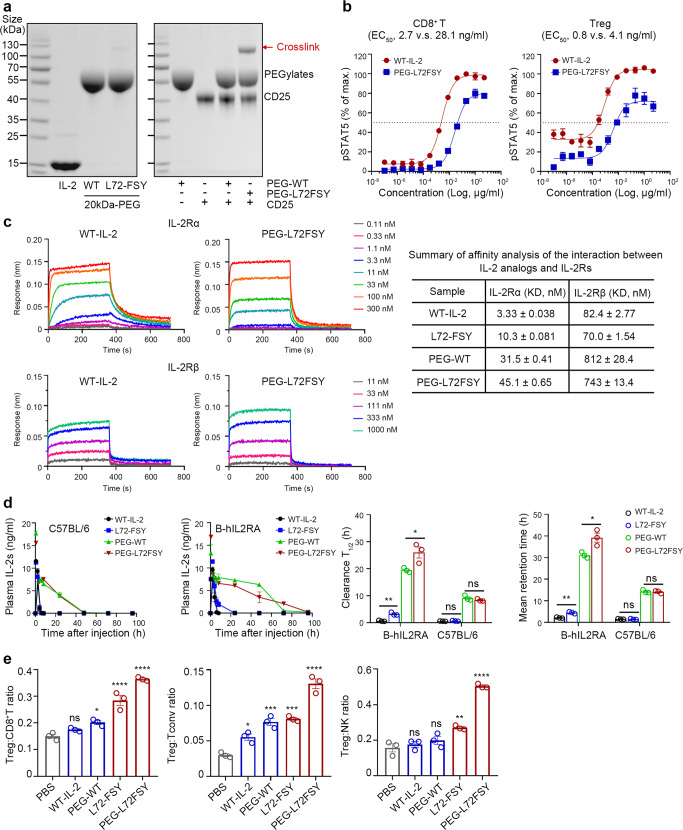
PEGylation of L72-FSY and characterization of the effects of PEGylated L72-FSY in vitro and in vivo. **a** N-terminal site-specific PEGylation of L72-FSY and its capacity to covalently bind to IL-2Rα were verified by Coomassie blue staining. IL-2 was incubated with a 5-fold molar excess of 20-kDa propionaldehyde polyethylene glycol (pALD-PEG) in the presence of sodium cyanoborohydride at pH 4.0 overnight. **b** Dose-dependent assessment of the ability of PEG-L72FSY or WT-IL-2 to activate Tregs and CD8^+^ T cells was performed by the pSTAT5 assay. hPBMCs from healthy donors were stimulated with the indicated samples in a series of 5-fold dilutions and then stained for quantitative flow analyses. The EC50 values of the above responses for the indicated cell populations were calculated and are shown. The symbol is plotted as the mean of triplicate wells, with the error bars representing the SEM derived from one donor, and the data are representative of three individual donors (*n* = 3). **c** Characterization of the binding affinities of the indicated samples for the extracellular domains of the α or β subunit of IL-2R by biolayer interferometry. The data are presented as the mean ± SD derived from indicated concentrations in a series of 3-fold dilutions. For panels **b**, **c**, all parameters are provided in Supplementary Table 3. **d** Characterization of the effects of PEGylation on the persistence of IL-2 circulation in two strains of mice. C57BL/6 and B-hIL2RA mice were subcutaneously injected with 5 µg of the indicated samples, and blood was collected at the indicated time points for quantification by ELISA. Bar graphs showing the comparisons of the persistence of the indicated samples in the circulation according to the calculated clearance half-life (T_1/2_) and mean retention time (MRT). All pharmacokinetics parameters are shown in Supplementary Table 4. **e** Validation of Treg-selective activation by PEG-L72FSY, as reflected by increased ratios of Treg:CD8^+^T, Treg:Tconv, and Treg:NK cells in treated B-hIL2RA mice. See also Supplementary Fig. 15. For panels (**d**, **e**), the data are presented as the mean ± SEM of three mice per group (*n* = 3), and representative results from one of two experiments are shown. The *p* values were determined by two-tailed unpaired Student’s t tests (panel **d**) and one-way ANOVA (Dunnett’s multiple-comparison test compared with PBS group, panel **e**), *p ≤ 0.05, **p ≤ 0.01, ***p ≤ 0.001, ****p ≤ 0.0001. The concentrations of PEGylated IL-2 reflect the molecular mass of IL-2 without the attached PEG

Our results demonstrated that L72-FSY preferentially and sustainably promoted the activation of Tregs over Teffs by covalently binding to IL-2Rα. In light of the inherent pharmacokinetic limit of IL-2, which is cleared very rapidly from the body due to its low molecular mass, we sought to determine the impact of prolonging retention in circulation on L72-FSY-mediated Treg-selective activation. We used a well-studied PEGylation strategy to extend the half-life of L72-FSY.^48^ The N-terminus of IL-2 was chosen as the PEGylation site due to its location at a suitable distance from receptor binding regions,^36^ thereby allowing the maintenance of IL-2 bioactivity after PEGylation. Incubation of WT-IL-2 or L72-FSY with 20-kDa propionaldehyde polyethylene glycol (pALD-PEG) at pH 4.0 overnight yielded a predominant mono-PEGylated product with a molecular weight of ~50 kDa. L72-FSY maintained its covalent binding activity after PEGylation (Fig. 5a). As expected, the bioactivity of PEGylated L72-FSY (PEG-L72FSY) was slightly decreased, whereas its selectivity was not affected, as demonstrated by the EC50 ratio of CD8^+^ T cells:Treg cells (Fig. 5b and Supplementary Table 3). A similar result was obtained in a parallel experiment using CD25-YT cells (Supplementary Fig. 14). These results were consistent with the affinity assessments, in which PEG-L72FSY exhibited mildly and equally decreased affinities for IL-2Rα and IL-2Rβ, showing effects on binding selectivity comparable to those of WT-IL-2 (Fig. 5c and Supplementary Table 3).

We next assessed the effects of PEGylation on IL-2 PK properties in two strains of mice after a single subcutaneous injection. As expected, the PK properties, including the clearance half-life (T_1/2_), blood concentration maintenance (AUC), and mean residence time (MRT), of both PEG-WT-IL-2 and PEG-L72FSY were markedly increased, suggesting that PEGylation reduced the in vivo clearance of IL-2s (Fig. 5d and Supplementary Table 4). To our surprise, the covalent binding of FSY further improved the PK properties of IL-2, as evidenced by observations that the parameters of FSY-bearing samples were obviously superior to those of their WT counterparts in B-hIL2RA mice but not those of C57BL/6 mice. Clearly, the marked improvement of PK properties in PEG-L72FSY was attributed not only to the increased molecular weight induced by PEGylation but also to the prolonged surface retainment that resulted from covalent binding. With the success of PEGylation of IL-2 at the N-terminus, we next examined its effects in vivo. A short-term and low-dose administration scheme was performed as previously reported.^17^ Briefly, B-hIL2RA mice were subcutaneously administered 0.5 μg of WT-IL-2 or L72-FSY for 10 consecutive days or an equal dose of their PEGylated forms once every other day for 2 weeks at 2-day intervals (Supplementary Fig. 15a). Strikingly, mobilization analysis of splenocytes (including murine Tregs, CD8^+^ T cells, Tconv cells and NK cells), which were harvested 5 days after the last injection, indicated that PEG-L72FSY induced the most preferential expansion of Tregs, followed by L72-FSY, as demonstrated by the elevated frequency of Tregs, but had no obvious impact on other effector cells (Supplementary Fig. 15b**)**. These selective effects resulted in increased Treg:CD8^+^ T-cell, Treg:Tconv-cell and Treg:NK-cell ratios that lasted for 5 days after the final administration (Fig. 5e). In contrast to PEG-L72FSY, PEGylated WT-IL-2 nonselectively increased the numbers of both Tregs and CD8^+^ T cells, which was consistent with the results of the binding affinity assessment, suggesting that N-terminal PEGylation has no impact on the pleiotropic effects of IL-2. Taken together, these data suggested that covalent binding synergizing with PEGylation, and specifically the resultant PEG-L72FSY, endows IL-2 with an enhanced and sustainable ability to preferentially promote the activation of Tregs, probably by preventing the rapid clearance of L72-FSY and thus increasing its chance for covalent binding.

### L72-FSY mitigated SLE and xeno-GvHD by promoting selective and persistent Treg proliferation

**Fig. 6:**
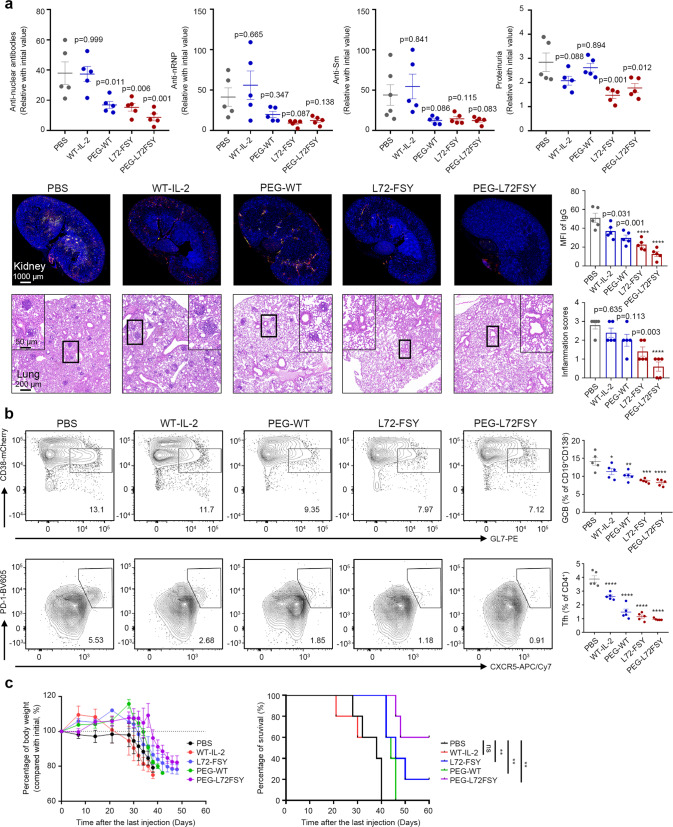
FSY-bearing IL-2 variants ameliorated SLE in a pristane-induced lupus model in B-hIL2RA mice and prevented GvHD aggravation. **a**, **b** B-hIL2RA mice were intraperitoneally injected with 0.5 ml of pristane. The establishment of lupus was confirmed by the detection of proteinuria two months later, and subcutaneous injection of the indicated samples at the indicated time points was then performed. The mice were sacrificed two weeks after the final injection for histopathological and immunohistochemical analyses. See also Supplementary Fig. 16-18. **a** The ability of FSY-bearing IL-2 variants to ameliorate signs of lupus, as demonstrated by reduced levels of proteinuria, serum anti-nuclear antibodies (ANAs), anti-ribonucleoprotein (rRNP) antibodies, anti-Smith (anti-Sm) antibodies, IgG and IgM deposition in the kidneys, and lymphocyte infiltration in the lung at the end of the experiment. Cryosections from the kidney were stained with anti-mouse IgG (red) and IgM (green), followed by analysis by fluorescence microscopy. IgG deposition was determined based on the MFI, which was calculated using ImageJ. The histological H&E inflammation score of the lungs, based on a 0-3 scale, was estimated according to the reported scoring criterion described in the supplementary methods section. **b** Representative flow plots and bar graphs reflecting the percentage changes in B cells with a GC phenotype (CD38^low^GL7^high^) and Tfh (CXCR5^+^PD^-^1^+^) cells among CD19^+^CD138^-^ B cells and CD4^+^ T cells, respectively, after the indicated treatment. **c** The effects of L72-FSY, WT-IL-2 and their PEGylated counterparts on a xeno-GvHD mouse model. A lethal dose of activated hPBMCs from healthy donors was injected into NSG mice, followed by subcutaneous injection of 1 μg of WT-IL-2 or FSY-72 for ten consecutive days or an equal dose of their PEGylated forms every other day five times. The weight curves and Kaplan–Meier survival curves of the grafted mice are shown. The data are presented as the mean ± SEM of 5 mice for panels **a**, **c** (*n* = 5), and the *p* values were determined by one-way ANOVA (Dunnett’s multiple-comparison test compared with the PBS group) for panels (**a**, **b**) and by the Mantel−Cox test for panel **c**. *p ≤ 0.05, **p ≤ 0.01, ***p ≤ 0.001, ****p ≤ 0.0001. The concentrations of PEGylated IL-2 reflect the molecular mass of IL-2 without the attached PEG

We next investigated the therapeutic potential of L72-FSY and its PEGylated form versus that of WT-IL-2 in a pristane-induced lupus model in B-hIL2RA mice as previously reported.^49^ The establishment of pristane-induced lupus was confirmed by the detection of proteinuria two months after pristane injection, and lupus mice were then randomly divided into five groups. Different forms of IL-2 were administered to the mice according to a long-term treatment schedule with slight change in which mice were treated with unmodified forms of IL-2s daily for 10 consecutive days or an equivalent dose of their PEGylated forms every other day for a total of five injections, and then maintenance treatment was performed every week for four additional injections^50^ (Supplementary Fig. 16a). Compared with WT-IL-2 and PBS treatment, L72-FSY and PEG-L72FSY treatment significantly ameliorated lupus disease severity, as determined by reduced levels of autoantibodies, including anti-nuclear antibodies (ANAs), anti-ribonucleoprotein (rRNP) antibodies and anti-Smith (anti-Sm) antibodies, as well as marked improvements in kidney damage, as evidenced by the amelioration of glomerulonephritis, renal inflammation, and tubular degeneration and decreases in immunoglobulin deposition and proteinuria (Fig. 6a and Supplementary Fig. 16b, c). Lung inflammation is another symptom of pristane-induced lupus mice that manifests as perivascular macrophage, neutrophil and lymphocyte infiltration.^50,51^ Notably, perivascular infiltration of mononuclear cells in the lung was readily observed in control mice but not L72-FSY- or PEG-L72FSY-treated mice (Fig. 6a), indicating that FSY-incorporated IL-2 suppressed lung inflammation in pristane-treated mice. Importantly, the IL-2-specific IgG levels in mice treated with the FSY-bearing IL-2 mutants during the long period of treatment were comparable to those in mice treated with WT-IL-2, suggesting that the incorporation of FSY at L72 and the resultant covalent binding did not introduce additional immunogenicity (Supplementary Fig. 16d).

Immunological and phenotypic analyses indicated that long-term treatment with L72-FSY and PEG-L72-FSY diminished the activity of Teffs to a greater extent than treatment with WT-IL-2, as evidenced by considerably decreased frequencies of CD8^+^ T and CD69^+^ Tconv cells, whereas no significant reduction in Tregs was observed in L72-FSY-treated mice compared with PBS-treated mice (Supplementary Fig. 17a). These effects correlated with increased ratios of splenic Tregs to CD8^+^ T cells and CD69^+^ Tconv cells as well as upregulated activation markers in Tregs but not CD8^+^ T and Tconv cells (Supplementary Fig. 17b). T follicular helper (Tfh) cells are another important type of Teff associated with the development and maintenance of germinal centres (GCs),^52^ and in agreement with published studies,^53^ WT-IL-2 treatment suppressed the differentiation of Tfh cells compared with PBS treatment. Notably, we found that long-term treatment with L72-FSY and PEG-L72-FSY induced a considerably greater reduction in the proportions of splenic Tfh cells and germinal centre B (GCB) cells than treatment with their WT counterparts or PBS treatment (Fig. 6b), resulting in remarkable reductions in splenic GC formation (Supplementary Fig. 17c). Concomitant with the inhibition of Tfh cells, we also found diminished proportions of T follicular regulatory (Tfr) cells, a cell type with properties of both Tregs and Tfh cells^54^ (CD3^+^CD4^+^Foxp3^+^CXCR5^+^Bcl-6^+^, Supplementary Fig. 18a), in the splenic Tregs of the L72-FSY- and PEG-L72FSY-treated mice relative to control mice (Supplementary Fig. 18b). Collectively, these data demonstrate the superiority of L72-FSY and PEG-L72-FSY in effectively suppressing immune responses and their therapeutic potential to ameliorate SLE disease activity and severity.

Finally, to explore therapeutic effects on human cells, we used a xenogeneic graft-versus-host disease (xeno-GvHD) mouse model in which IL-2-expanded Tregs prevent GvHD by suppressing the proliferation of injected hPBMCs.^17^ Briefly, immunodeficient NSG mice were injected with a lethal dose of hPBMCs from one healthy donor and then administered 1 μg of WT-IL-2 or FSY-72 for ten consecutive days or an equal dose of PEG-WT or PEG-L72FSY every other day five times. Consistent with the results from the pristane-induced model, mice that were administered FSY-bearing IL-2 variants were more protected from GvHD than control mice that were injected with PBS or WT-IL-2. PEG-L72FSY provided the most protection of all the samples tested, followed by its non-PEGylated counterpart, as evidenced by reduced body weight loss and increased survival rates, and such protective effects lasted for more than 8 weeks (Fig. 6c). Collectively, these data suggest that L72-FSY and its long-acting form are superior to WT-IL-2, can selectively induce Tregs in pristane-induced lupus mice, and are more effective in mitigating inflammatory diseases such as SLE and GvHD.

## Discussion

LD-IL-2 has been used as an immunosuppressive treatment for a number of ADs, but its clinical benefits are limited due to its paradoxical effects and short half-life, which makes its therapeutic window as an immunosuppressant rather narrow.^7,9^ Upon engagement, IL-2−receptor complexes on the cell surface membrane are internalized into endosomes, where IL-2 can either be routed for degradation in lysosomes with IL-2Rβ and γ_c_, resulting in signal attenuation, or be recycled back to the cell surface with IL-2Rα.^22,23^ Modulating intracellular trafficking represents a promising strategy for enhancing the potency and selectivity of IL-2.^1,2,5^ Although high-affinity protein mutants can evolve to promote tighter binding, such binding remains noncovalent and reversible in nature, making attempts to generate Treg-selective agonists elusive.

In this study, by using a latent bioreactive artificial amino acid incorporated by genetic code expansion,^33,34^ we successfully developed an IL-2 variant with a more potent binding affinity for IL-2Rα than that attained by natural amino acid mutagenesis. Our results demonstrated that these covalent IL-2 mutants, exemplified by L72-FSY, considerably promoted the activation of Tregs by profoundly increasing the recycling of IL-2 and selectively enhancing signalling potency, thereby skewing the immune balance towards suppression in a sustained manner.

Evaluation of the covalent binding ability to IL-2Rα showed that FSY enabled proximity-induced reactivity when it was close enough to the natural target residue to promote ligand–receptor interactions, and the crosslinking efficiency was largely dependent on the site at which FSY was incorporated. No obvious crosslinking was observed for either R38- or F42-FSY despite the accessibility of their crystal structures, and no covalent crosslinking of L72-FSY to other binding proteins, such as mIL-2Rα, was detected, verifying the high specificity and selectivity of FSY-mediated crosslinking. Furthermore, we found that the crosslinking efficiency was strictly dependent on the temperature and time of the reaction, as the overall efficiencies overnight at 37 °C were tenfold greater than those at 4 °C. We hypothesize that a balance may exist between crosslinking efficiency and specificity, as an amino acid group with more activity may improve the crosslinking efficiency but reduce the specificity of the reaction.

Although a covalent linkage between IL-2 and IL-2Rα was achieved, we found that the affinity of L72-FSY for IL-2Rα was comparable or slightly decreased compared with that of WT-IL-2. The decrease of affinity is reasonable since L72 of IL-2 participates in binding with IL-2Rα,^35,36^ and the duration of covalent bond formation is longer than that of dissociation on the affinity assessment using BLI. Thus, a covalent binding strategy with slightly compromised association capacity has been exploited to develop competitive antagonists to inhibit endogenous pathogenic cytokines regardless of binding affinity.^34^ Our study was the first to successfully develop an IL-2 variant as a potent and selective agonist but not antagonist of Tregs by enhancing IL-2 recycling with covalent binding. The general approach described herein provides a direction for engineering cytokines, especially for those that could be improved by modulating cellular trafficking.

Notably, L72-FSY promoted the proliferation and expansion of MDSCs in IL-2Rα-humanized mice in vivo. MDSCs are a heterogeneous population of cells with immunosuppressive effects that have been demonstrated to promote cancer progression in humans by suppressing antitumour T cells.^43^ To date, MDSC accumulation has been documented in a number of ADs,^55–57^ although the roles of MDSCs in the pathogenesis of ADs remain elusive.^58^ The elevated levels of MDSCs induced by L72-FSY were unlikely to be due to direct activation caused by covalent binding, as IL-2Rα is absent in all types of MDSCs. Indirect factors, such as potential immunoregulatory crosstalk via the production of cytokines,^43^ may promote the expansion of MDSCs. Further investigations of the underlying mechanisms and the therapeutic implications of MDSCs are warranted.

Although inhibitory receptors, including CTLA-4, PD-1, LAG-3 and Tim-3, have been extensively investigated in the context of Teff dysregulation and exhaustion, their impact on Tregs is not well known.^59^ LAG-3 intrinsically limits Treg proliferation and function at inflammatory sites,^46^ while other inhibitory molecules, particularly PD-1, are generally associated with enhanced Treg functions.^45,60^ Compared with WT-IL-2, exogenous L72-FSY induced Tregs to express less LAG-3 and simultaneously enhanced the expression of PD-1 and Tim-3. This result correlated well with the superior effects of L72-FSY in promoting the activation and proliferation of Tregs relative to its WT counterparts both in vitro and in vivo. Of note, in contrast to WT-IL-2, L72-FSY notably promoted the expression of CD62L with less production of IL-10 in response to stimulation, providing mechanistic evidence that L72-FSY maintains Tregs in a central memory phenotype without driving terminal differentiation and may enhance the homing properties of Tregs that migrate to lymphoid tissue.^44,61^ This intriguing effect of L72-FSY was similar to that in a recent report on an engineered IL-2 partial agonist that has the capacity to promote CD8^+^ T-cell stemness and could also account for the superior therapeutic value of L72-FSY relative to its WT counterparts.^62^ Given that the effects of WT-IL-2 leads to the differentiation of T cells into an effector phenotype and lowers their therapeutic efficacy,^63,64^ whether L72-FSY and its PEGylated counterparts could synergize with adoptive Treg therapy and the detailed impacts on downstream signalling warrant further investigation.

Given the long period of disease progression, we employed a long-term administration scheme in a pristane-induced lupus model, which revealed that L72-FSY and PEG-L72FSY diminished the activity of Teffs to a greater extent than treatment with WT-IL-2, as demonstrated by the considerably decreased frequencies of CD8^+^ T and CD69^+^ Tconv cells. This result was consistent with a previous study demonstrating that long-term WT-IL-2 treatment resulted in the clinical and histological amelioration of lupus nephritis by reducing the activity and proliferation of both Tconv cells and Tregs.^50^ The decreases in hyperactivity and the number of Teff cells were probably due to the immunoregulatory effects of Tregs and associated with the progression of disease, which led to a compensatory reduction in the number of Tregs. Notably, mice treated with L72-FSY or PEG-L72-FSY exhibited higher levels of Tregs than their WT counterparts, resulting in elevated ratios of Tregs:CD8^+^T cells and Tregs:CD69^+^Tconv cells, and this selective effect lasted for at least two weeks after the final injection. The sustained effects of L72-FSY on Tregs were consistent with the results of functional and phenotype assays, which revealed that L72-FSY maintained Tregs in a central memory phenotype without driving terminal differentiation. Collectively, these results support that long-term administration of L72-FSY or PEG-L72FSY is capable of ameliorating the progression of ADs, and the effects of L72-FSY and PEG-L72FSY were superior to those of WT-IL-2 treatment.

Despite the superiority of L72-FSY over WT-IL-2 in terms of Treg selectivity, repeated injection of an L72-FSY bolus at a sufficient dose is still required to ensure sufficient receptor occupancy on Tregs, because of its rapid in vivo clearance. To improve and prolong the in vivo circulatory persistence of L72-FSY, we used a 20-kDa PEGylation strategy, which significantly improved the PK properties without significantly compromising bioactivity. Indeed, we demonstrated that PEG-L72FSY was better than its parental form at preferentially expanding Tregs and required less frequent injections, resulting in a sustained skewing of the immune balance towards suppression and the alleviation of ADs. These findings slightly conflict with the previous viewpoint that the irreversible binding of protein drugs obviates the requirement of half-life extension.^34^ The improved Treg-selective activation by PEG-L72FSY over L72-FSY might be explained by several factors, such as the decreased binding affinity for IL-2Rβ-γ_c_ and increased occupancy of IL-2 on Tregs within the time frame of metabolic clearance. Intriguingly, we also found that the treatment efficacy of the PEGylated agents in lupus mice was generally better than that of the non-PEGylated agents, which was most likely due to their improved PK properties and underscores the importance of protein modifications. The modified cellular trafficking and prolonged half-life of PEG-L72FSY make it a promising therapeutic option for ADs due to its preferential, enhanced and sustainable effects on Treg cells.

The N-terminus of IL-2 was selected as the PEGylation site because of its accessibility for modification under mildly acidic conditions^48^ as well as its structurally distant location away from receptor binding regions.^35,36^ Recently, we successfully developed a series of site-specific PEGylated IL-2 mutants with preferential binding of the IL-2Rα subunit over the IL-2Rβ subunit, leading to sustained activation of Tregs.^19^ The precision PEGylation strategy involved genetic code expansion-guided incorporation of azide-bearing amino acids and the subsequent orthogonal conjugation of PEG moieties. The combination of such site-specific PEGylation with covalent binding is desirable for improving the selectivity and PK properties of IL-2, but this strategy remains challenging due to multi-incorporation of UAAs and unsatisfactory production yields. A number of techniques, including optimizing the incorporation systems containing orthogonal tRNA synthetase and tRNA pairs, conditional knockout of release factor 1, and unassigned codon utilization, might prove to be promising and feasible in future explorations.^65,66^

One limitation of this study is the lack of therapeutic results for L72-FSY/PEG-L72FSY in various AD models, particularly in spontaneous disease models due to the limitation of humanized AD mouse model. A potential issue associated with the clinical application of IL-2Rα that covalently binds to IL-2 for the treatment of AD is the ability of Teffs to upregulate IL-2Rα,^6,18^ although this usually requires robust activation. In addition, the expression levels of IL-2Rα in Teffs are generally lower than those in Tregs after stimulation.^64^ Optimizing the treatment regimen, including the dose, administration frequency and timing of administration, is important for improving efficacy and minimizing potential side effects.

Collectively, our results show that the covalent binding of IL-2 to IL-2Rα prolongs the engagement of IL-2 on Tregs with enhanced potency. The IL-2 variant developed in this study, which preferentially activates and expands Tregs in a highly specific and persistent manner for an extended duration, is effective in treating inflammatory disease, including SLE and GvHD. This study thus represents a promising direction for the development of clinically amenable IL-2 therapies for AD treatment. Furthermore, the general approach to covalent bond formation presented in this study may enable the development of other pivotal cytokine mutants with enhanced potencies, thereby exerting profound effects on their therapeutic potencies via modulation of cellular trafficking.

## Materials and methods

### Ethics approval statements

All animal experiments were conducted in accordance with the National Institute of Health Guide for Care and Use of Laboratory Animals with the approval of the Ethics Review Board of PUMC Hospital, Chinese Academy of Medical Science (CAMS) (Beijing, China).

### Crosslinking of IL-2 (FSY) with IL-2Rα in vitro

Purified WT-IL-2 or FSY-bearing IL-2 variants were incubated with human IL-2Rα (*SinoBiological, Inc., China*) at a molar ratio of 1:1 in D-PBS at 37 °C overnight. After incubation, 5 × loading buffer was added, and the samples were heated at 99 °C for 10 min to stop the reaction. The samples were analysed on a 12% SDS-PAGE gel followed by Coomassie Blue staining under reducing conditions. In parallel, western blot analysis was performed using a primary anti-6 × His-tag antibody (*Thermo, USA*) at a 1:3000 dilution and a secondary HRP-conjugated goat anti-mouse IgG antibody (*Thermo, USA*). Kinetic analysis of the crosslinking reaction of IL-2(FSY)/IL-2Rα was determined by incubating L72-FSY with human IL-2Rα at a molar ratio of 1:1 at 37, 25 and 4 °C for different time amounts of time, and the samples were analysed with SDS-PAGE followed by Coomassie Blue staining as described above.

To assess the crosslinking of IL-2 (FSY) with IL-2Rα on cells, CD25-engineered YT cells were resuspended at 1 × 10^6^/ml in complete medium (RPMI 1640, 10% FBS, 1:100 NEAA and penicillin-streptomycin) and then aliquoted into 24-well plates at 500 µl/well (5 × 10^5^). Equal amounts of protein at different concentrations were suspended in complete medium and added to the test wells, and the cells were cultured at 37 °C for the indicated amounts of time. The cells were then collected and lysed by the addition of 200 μL of RIPA buffer containing a protease inhibitor cocktail (*Roche Inc., USA*). After centrifugation, the protein concentration in the supernatant was quantified using a BCA protein assay kit (*Thermo Inc., USA*). Equivalent amounts of protein samples were then heated at 99 °C for 10 min with 5 × loading buffer, separated by 12% SDS-PAGE, and subjected to western blot using a primary anti-IL-2Rα antibody (No. 231441, *Abcam, USA*), the internal standard anti-GAPDH, and a secondary HRP-conjugated goat anti-rabbit or mouse IgG antibody. The protein bands were detected by chemiluminescence.

### In vitro recycling assays of isolated human Treg and CD8^+^ T cells

The in vitro recycling assays were performed as previously reported with slight changes.^24^ Briefly, to obtain purified Treg cells, hPBMCs were isolated from healthy donors by Ficoll. The CD4^+^ T cells were first enriched by negative selection with magnetic-activated cell sorting according to the manufacturer’s directions, and Tregs (CD4^+^ CD25^high^ CD127^low^) were then sorted on a FACSAria instrument (*BD Biosciences*). The sorted Tregs were expanded with IL-2 and CD3/CD28 beads ex vivo for 9 days and allowed to rest at 37 °C in culture medium (RPMI 1640, 10% FBS, 1:100 penicillin-streptomycin) for one day prior to the assay. CD8^+^ T cells were isolated from healthy donors by negative selection according to the manufacturer’s instructions and used immediately.

For the recycling assays, resting Treg cells or CD8^+^ T cells were incubated with 200 ng/ml IL-2 or L72-FSY at 37 °C for 1.5 h. The cultured cells were then washed with acid wash buffer (assay medium adjusted to pH 3.5 with HCl) three times and replated in assay medium at 37 °C for the indicated amount of time without cytokines. For the analysis of IL-2 or L72-FSY recycling to the cell surface, live cells were washed and directly stained with an Alexa Fluor 647 (AF647)-labelled anti-IL-2 antibody for flow cytometry analysis. For pSTAT5 assays, the cultured cells were mixed with fixation buffer (*BD Biosciences*) at a 1:1 ratio for 15 min at 37 °C. After washing three times with D-PBS, the cells were pelleted by centrifugation and then resuspended in permeabilization buffer III (*BD Biosciences*) for 15 min on ice. The fixed and permeabilized cells were washed twice with FACS buffer and stained with an anti-human pSTAT5(pY694)-Pacific Blue antibody.

All FACS antibodies were used at a 1:50 dilution. Data were collected on a CytoFLEX instrument (*Beckman Coulter Inc., USA*) and analysed using CytExpert 2.4 and FlowJo software. The background was defined as the mean fluorescence intensity (MFI) of anti-IL-2-AF647 or anti-human pSTAT5 Pacific Blue in non-IL-2-treated but stained cells, and the data were plotted as the background-subtracted MFI normalized to the maximum for each treatment.

### Confocal microscopy

For localization analysis of IL-2 and L72-FSY upon binding to IL-2Rα, Alexa Fluor 555 (AF555, *Thermo, USA*)-labelled samples (200 ng/ml) were incubated with CD25-YT cells for 6 h at 37 °C. Surface and endocytic localization was then revealed by sequentially incubating the cells with a mouse anti-human CD25 antibody (*Thermo, USA*) and then with AF647-labelled anti-mouse IgG (*Biolegend, USA*) and LysoTracker Blue BND-22 (*Thermo, USA*) at a 1:5000 dilution for 30 min in the dark. The samples were rinsed twice with PBS supplemented with 3% FBS between each step. The cells were then imaged using a confocal laser-scanning microscope (Nikon, Tokyo, Japan).

### Assay of in vitro and in vivo cell proliferation and IL-2-induced protein expression

The L72-FSY-mediated proliferation and protein expression of specific cell subsets in vitro was analysed as previously described with slight changes.^17^ Briefly, hPBMCs were isolated from healthy and patient donors by Ficoll and resuspended in complete medium (RPMI 1640, 10% FBS, sodium pyruvate, nonessential amino acid (NEAA) and penicillin/streptomycin). Then, 100 µl/well (2 × 10^5^) aliquots of resuspended cells were distributed into a 96-well V-bottom plate, and an equal volume of 2 × WT-IL-2 or L72-FSY was added to the test wells in serial 5-fold dilutions. The cells were incubated with the samples at the indicated concentrations for 6 h, washed with acid buffer, and then cultured without cytokines for three days. PBS-treated cells were used as a negative control. After incubation, the cultured cells were centrifuged, resuspended in FACS buffer, and stained extracellularly with anti-human CD3-APC/Cy7, CD4-FITC, CD8-BV510, CD16-percp5.5, CD56-percp5.5, CD25-APC, CD49d-BV605, CD25-PE, and CD69-BV785 antibodies. The cells were then washed twice with D-PBS containing 0.2% BSA and fixed with Foxp3 Transcription Factor Fixation/Permeabilization Buffer (eBioscience) for 30 min on ice. After two washes with permeabilization buffer (eBioscience), the samples were intracellularly stained with anti-human Foxp3-PB for 1 h at room temperature.

To assay the responses of human immune cells to FSY-bearing IL-2 in vivo, a humanized NOD-SCID IL-2 receptor gamma null (NSG) mouse expansion model was utilized as described previously with slight changes.^17^ Briefly, hPBMCs were isolated from single healthy donors by Ficoll and injected intravenously (5 × 10^6^) into 8- to 10-week-old NSG mice. The injected mice (four per group) were then subcutaneously injected with IL-2 or L72-FSY daily for ten consecutive days for a total of ten times or with their PEGylated forms every other day for a total of five times. The mice were sacrificed by cervical dislocation five days after the last injection, and their splenocytes were collected for flow cytometry analysis. The samples were extracellularly stained with anti-human CD45-PB, CD3-APC/Cy7, CD4-FITC, CD8-BV510, and CD25-APC and intracellularly stained with anti-human Foxp3-PE using the Foxp3 Buffer Kit (eBioscience) according to the manufacturer’s directions. For determination of cell counts based on flow cytometry, the samples were analyzed on flow cytometry in a fixed recorded events, and events were normalized to counting beads with known concentration (provided by manufacturer) at each assessment. The absolute numbers of cells were calculated as (recorded cell events/recorded beads events) × (number of beads in tube) and multiplied by the dilution factor.

All FACS antibodies were used at a 1:50 dilution. The stained cell suspensions were filtered through a 70 mm nylon cell strainer (BD Biosciences), and equal numbers of cells were analysed by flow cytometry to assess their proliferation and protein expression. Data were collected on a CytoFLEX instrument (Beckman Coulter Inc., USA) and analysed using CytExpert 2.4 and FlowJo software. The Animal Use Protocol was reviewed and approved annually by the Ethics Review Board of PUMC Hospital, Chinese Academy of Medical Science (CAMS).

### CyTOF analysis of L72-FSY in mouse immune cells in vivo

To assess the effects of IL-2 and L72-FSY on mouse immune cells in vivo, IL-2Rα-humanized B6 (B-hIL2RA) mice (*Biocytogen Inc. Beijing*), in which the extracellular domain of IL-2Rα was humanized, were employed. Briefly, B-hIL2RA mice (8-10 weeks old, three per group) were subcutaneously injected with IL-2 or L72-FSY daily for ten consecutive days for a total of ten times. The treated mice were sacrificed at the indicated time point after the last injection, and their splenocytes were collected for flow cytometry analysis. The FACS antibodies used included anti-mouse CD3-BV510, CD4-PE/Cy7, CD8-BV605, Foxp3-PB, CD49b-PE, CD11b-PerCP/Cyanine5.5, Ly-6G-PE/Dazzle594, Ly-6C-BV785 and anti-human CD25-BV650. For the analysis of IL-17- and IFNγ-producing cells, splenocytes were stimulated with a 1 × cell stimulation cocktail (plus protein transport inhibitors) for 4 h and then extracellularly stained with anti-human CD3 and CD4 antibodies and intracellularly stained with anti-mouse IL-17A-PE and IFNγ-BV421 antibodies as described above.

Half of the spleens were collected on day 5 after the last injection for mass cytometry analysis. CyTOF analyses were performed by PLTTech Inc. (Hangzhou, China) according to a previously described protocol.^67^ Briefly, the collected spleens were dissociated into single cells with DNase and Liberase and enriched by Percol. Qualified samples were blocked and stained for 30 min with a surface antibody panel designed in house (Supplementary Table 2), followed by fixation overnight. The next day, the fixed cells were washed, permeabilized and incubated with an intracellular antibody mixture. The rinsed cells were analysed using a CyTOF system (*Helios, Fluidigm, South San Francisco, CA, USA*), and the types of immune cells were identified via nonlinear dimensionality reduction (t-distributed stochastic neighbour embedding, tSNE), followed by density clustering.

### N-terminal PEGylation and purification of WT-IL-2 and L72-FSY by propionaldehyde polyethylene glycol

N-terminal PEGylation and protein purification were performed as previously reported with slight changes.^19,68^ Briefly, WT-IL-2 and L72-FSY were reacted with 20-kDa pALD-PEG at a 1:5 molar ratio in 20 mM sodium acetate and 10 mM sodium cyanoborohydride, pH 4.0. The PEGylation reaction was conducted at 25 °C overnight. The reacted sample was diluted to an appropriate concentration while adjusting the pH adjusted to 4.5 with glacial acetic acid, and the samples was then loaded onto a SOURCE 15 S column equilibrated with 20 mM sodium acetate, pH 4.5. PEGylated IL-2s were eluted with a 4–35% linear gradient of elution buffer (20 mM sodium acetate, 2 M NaCl, pH 4.5, in 15 column volumes). The main elution peak was pooled, concentrated, and buffer exchanged into D-PBS by ultrafiltrationat least three times. The eluted PEGylated IL-2 was further on a Superdex 200 increase column, with 20 mM Tris-HCl and 500 mM NaCl, pH 8.0, serving as the running buffer. The main elution peak was collected, concentrated, and frozen at −80 °C until use. PEGylated IL-2 was confirmed by SDS–PAGE and staining with Coomassie Blue dye. The IL-2 and PEGylated IL-2 concentration were measured using the bicinchoninic acid (BCA) protein assay (Pierce). Since the PEG moiety have no response to BCA method, the concentrations of PEGylated IL-2 reflect the molecular mass of IL-2 without the attached PEG.^19^

### Assaying the effects of L72-FSY-IL-2 on mice with pristane-induced lupus mice

The pristane-induced lupus model was employed to evaluate the abilities of these samples to alleviate SLE disease activity after the intraperitoneal injection of 0.5 ml of pristane on day 0 into female B-hIL2RA mice. The establishment of lupus was confirmed by the detection of proteinuria two months later, and lupus-positive mice were randomly divided into five groups for subsequent assessment. An established long-term IL-2 treatment schedule was employed with slight changes.^50^ Briefly, the resulting lupus-positive mice were subcutaneously injected with 1 μg of WT-IL-2 or L72-FSY for ten consecutive days or with an equal dose of their PEGylated forms every other day for total of five injections. Treatment was maintained for the mice in all groups by the repetitive injection of the corresponding samples every week for one month (four additional injections). The mice were bled and placed into individual metabolic cages for urine collection and sacrificed two weeks after the final injection. Their kidneys, spleens, lungs and plasma were excised for analysis. ANAs, anti-nRNP antibodies and anti-Sm antibodies were detected by ELISA kits (*Alpha Diagnostic International, USA*) according to the manufacturer’s instructions, and proteinuria levels were determined by the Bradford (*Takara, Inc*.) method for protein quantitation. The baseline levels of autoantibodies and proteinuria were determined prior to sample administration. At the end of the experiment, all mice were sacrificed by cervical dislocation, and their kidneys, lymph nodes, spleens and plasma were excised for histopathological and immunofluorescence (IF) examinations.

### Antibodies used for flow cytometry experiments

The following antibodies were used to stain human cells: CD45 Pacific Blue (clone HI30, Biolegend, no. 304022), CD3 APC/Cyanine7 (clone UCHT1, Biolegend, no. 300426), CD3 Brilliant Violet 510 (clone UCHT1, Biolegend, no. 300448), CD4 PE/Cyanine7 (clone RPA-T4, Biolegend, no. 300512), CD4 FITC (clone RPA-T4, Biolegend, no. 317408), CD8 Brilliant Violet 510 (clone RPA-T8, Biolegend, no. 301048), CD8 Brilliant Violet 785 (clone SK1, Biolegend, no. 3447040), CD8 FITC (clone SK1, Biolegend, no. 344704), CD25 APC (clone BC96, Biolegend, no. 302610), CD25 Brilliant Violet 650 (clone BC96, Biolegend, no. 302634), Foxp3 PE (clone 206D, Biolegend, no. 320108), Foxp3 Pacific Blue (clone 206D, Biolegend, no. 320116), CD127 PE (clone A019D5, Biolegend, no. 351304), CD16 Brilliant Violet 605 (clone 3G8, Biolegend, no. 302040), CD56 Brilliant Violet 605 (clone HCD56, Biolegend, no. 318334), CD16 PerCP/Cyanine5.5 (clone 3G8, Biolegend, no. 302028), CD56 PerCP/Cyanine5.5 (clone HCD56, Biolegend, no. 318322), CD49d Brilliant Violet 605 (clone 9F10, Biolegend, no. 304324), IL-17A PE (clone BL168, Biolegend, no. 512306), IFN-γ Brilliant Violet 510 (clone 4 S.B3, Biolegend, no. 502544), pSTAT5 Pacific Blue (clone 47/Stat5(pY694), BD Biosciences, no. 560311), CD69 Brilliant Violet 785 (clone FN50, Biolegend, no. 310932), IL-2 Brilliant Violet 421 (clone MQ1-17H12, Biolegend, no. 500328), Anti-IL-2 Receptor alpha (clone SP176, abcam, no. ab231441), Anti-6xHis tag (Clone HIS.H8, abcam, no. ab18184), CD44 FITC (clone BJ18, Biolegend, no. 338804), CD62L APC (clone DREG-56, Biolegend, no. 304810), CD279 Brilliant Violet 650 (clone EH12.2H7, Biolegend, no. 329950), CD366 PE/Cyanine7 (clone F38-2E2, Biolegend, no. 345014), CD233 APC (clone 7H2C65, Biolegend, no. 369212), CD152 Brilliant Violet 785 (clone BNI3, Biolegend, no. 369624), Helios FITC (clone 22F6, Biolegend, no. 137214), CD278 PE (clone C398.4 A, Biolegend, no. 313508), IL-10 PE/Dazzle594 (clone JES3-19F1, Biolegend, no. 506812), LAP PE (clone S20006A, Biolegend, no. 300004).

The following antibodies were used to stain mouse cells:

CD3 APC/Cyanine7 (clone 17A2, Biolegend, no. 100222), CD3 Brilliant Violet 510 (clone 17A2, Biolegend, no. 100234), CD4 PE/Cy7 (clone RM4-5, Biolegend, no. 100528), CD8a FITC (clone 53-6.7, Biolegend, no. 100706), CD8a Brilliant Violet 605 (clone 53-6.7, Biolegend, no. 100744), Foxp3 Pacific Blue (clone MF-14, Biolegend, no. 126410), Foxp3 Brilliant Violet 421 (clone MF-14, Biolegend, no. 126419), CD49b (pan-NK cells) PE (clone DX5, Biolegend, no. 108908), CD69 APC/Cyanine7 (clone H1.2F3, Biolegend, no. 104526), Ly-6G PE/Dazzle 594 (clone 1A8, Biolegend, no. 127648), Ly-6C Brilliant Violet 785 (clone HK1.4, Biolegend, no. 128041), CD11b PerCP/Cyanine5.5 (clone M1/70, Biolegend, no. 101228), IL-17A PE (clone TC11-18H10.1, Biolegend, no. 506904), IFN-γ Brilliant Violet 421 (clone XMG1.2, Biolegend, no. 505830), IgM mu chain Alexa Fluor 647 (abcam, ab150123), IgG Fc FITC (abcam, ab97264), C3 (clone 11H9, abcam, ab11862), CD19 PE/Cy7 (clone 6D5, Biolegend, no. 115520), CD138 Brilliant Violet 510 (clone 281-2, Biolegend, no. 142521), CD38 PE/Dazzle594 (clone 90, Biolegend, no. 102730), GL7 PE (clone GL7, Biolegend, no. 144608), Bcl-6 Percp/Cyanine5.5 (clone 7D1, Biolegend, no. 358508), PD-1 Brilliant Violet 605 (clone 29 F.1A12, Biolegend, no. 135219), CXCR5 APC/Cyanine7 (clone L138D7, Biolegend, no. 145526).

All FACs antibodies were used at a 1:50 dilution.

### Statistical analysis

All statistical calculations were performed using GraphPad Prism 8.0. Accordingly, data sets were subjected to one-way ANOVA (Dunnett’s multiple comparison), the two-tailed paired or unpaired Student’s t-test, and the Mantel-Cox test as indicated in the figures. p values less than 0.05 were considered significant. *p ≤ 0.05, **p ≤ 0.01, ***p ≤ 0.001, ****p ≤ 0.0001.

## Supplementary information

SUPPLEMENTAL MATERIAL

## Data Availability

The main data supporting the results in this study are available within the paper and its Supplementary Information. The raw and analysed datasets generated during the study are too large to be publicly shared, yet they are available for research purposes from the corresponding authors on reasonable request. All unique materials, such as unnatural amino acid, plasmid and IL-2 variants are readily available from authors. The quaternary structure of IL-2 assembled to the trimeric receptor is available from publicly available datasets (PDB number: 2ERJ, http://www1.rcsb.org).
