# Supplementary material for: Proximity-enabled covalent binding of IL-2 to IL-2Rα selectively activates regulatory T cells and suppresses autoimmunity
Source: Signal Transduct Target Ther. 2023 Jan 23;8:28. doi: 10.1038/s41392-022-01208-3 (PMC9871032; doi:10.1038/s41392-022-01208-3)
Supplement: Supplementary file 1 — SUPPLEMENTAL MATERIAL [file 41392_2022_1208_MOESM1_ESM.docx]

Supplementary Materials for

**Proximity-enabled covalent binding of IL-2 to IL-2Rα selectively and persistently activates Treg and suppresses autoimmunity**

Bo Zhang^1,2†*^, Jiaqi Sun^2†^, Yeshuang Yuan^3†^, Dezhong Ji^2†^, Yeting Sun^1,4†^, Yudong Liu^3^, Shengjie Li^1^, Xingxing Zhu^2^, Xunyao Wu^1^, Jin Hu^1^, Qiu Xie^1^, Ling Wu^2^, Lulu Liu^1^, Boyang Cheng^2^, Yuanjie Zhang^2^, Lingjuan Jiang^1^, Lidan Zhao^4^, Fei Yu^2^, Wei Song^1^, Min Wang^3^, Yue Xu^3^, Shiliang Ma^3^, Yunyun Fei^1,4^, Lihe Zhang^2^, Demin Zhou^2*^ and Xuan Zhang^3,1*^

Correspondence to: [zxpumch2003@sina.com](mailto:zxpumch2003@sina.com); [deminzhou@bjmu.edu.cn](mailto:deminzhou@bjmu.edu.cn); zhangbo98@pumch.cn

**This PDF file includes:**

Materials and Methods

Figures. S1 to S18

Tables S1 to S4

Materials and Methods

**General materials**

The DH5α strain was used to clone and propagate plasmid DNA. Miniprep and Maxiprep Kits (*Axygen, USA*) were used to harvest and purify plasmid DNA. A QuikChange Lightning Site-Directed Mutagenesis Kit (*Agilent, USA*) was used to generate site-directed mutations. The unnatural amino acid FSY was synthesized using the SO_2_F_2_/borax method as previously reported^1^. Briefly, Boc-Tyr-OH was added to a saturated Borax solution, and then the reaction system was vacuumed and refilled with SO_2_F_2_. The reaction mixture was stirred vigorously at 25°C overnight to obtain Boc-Tyr-OSO_2_F. Then, Boc-Tyr-OSO_2_F was treated with 4 M HCl in dioxane, and the reaction mixture was stirred overnight. The precipitated solid was filtered and washed with cool ether, producing the targeted fluorosulfate-L-tyrosine HCl salt as a white solid. Twenty-kDa propionaldehyde polyethylene glycol (20-kDapALD-PEG, *Aladdin, Inc., Shanghai*) was used to perform N-terminal PEGylation.

**Expression and purification of wild-type and FSY-containing human IL-2-His×6variants**

The cDNAs encoding human IL-2 were cloned into the pET-21a (+) expression vector (Novagen) containing the T7 promoter, a 6× His tag at the C-terminus, and ampR. The plasmid harbouring the IL-2 gene was mutated such that the residue targeted for unnatural amino acid incorporation was replaced with an amber stop condon (TAG). The obtained plasmids were transformed into the OrigamiB (DE3) strain either with or without pEVOL-FSY, which contained the expanded genetic code system for an amber codon-suppressing tRNA Pyl under the control of the proK promoter and FSY-specific *Methanosarcina mazei*-derived aminoacyl-tRNA synthetase^1^ under the control of the TacI promoter. For the incorporation of FSY into IL-2, briefly, transformed OrigamiB (DE3) cells were grown in 2× YT medium with 100 μg/mL ampicillin and 50μg/mL spectinomycin and induced with 0.5 mM IPTG and 0.5 mM FSY when the OD 600 reached approximately 0.8-1.0. After overnight induction at 20 °C, all cell samples were harvested by centrifugation and resuspended in precooled His-binding buffer (20 mM Tris-HCl, pH 8.0, 250 mM NaCl, 5 mM imidazole). Protein extraction was performed by passing cells through a microfluidizer twice at 1200 bar while cooling, and the supernatant was collected by centrifugation.

For the purification of IL-2 and IL-2 variants, an appropriate amount of Ni-NTA agarose beads (*Thermo, USA*) was added to the supernatant and incubated at 4 °C for 2 hours while shaking. The mixture was loaded onto a column and washed with more than ten column volumes of wash buffer (20 mM Tris-HCl, pH 8.0, 500 mM NaCl, 20 mM imidazole). The protein was eluted with elution buffer (20 mM Tris-HCl, pH 7.6, 250 mM NaCl, 250 mM imidazole) and quickly buffer exchanged into D-PBS by [ultrafiltration](file:///D:\\协和课题\\IL-2靶向激活\\论文用\\science%2520trans%2520med\\javascript:;) (*Millipore, USA*) two times. Next, the concentrated sample was diluted to an appropriate concentration while adjusting the pH adjusted to 4.5 with glacial acetic acid, and the sample was then loaded onto a SOURCE 15S (*GE Healthcare*) column equilibrated with 20 mM sodium acetate, pH 4.5. IL-2 and the IL-2 variants were eluted with a 10-40% linear gradient of elution buffer (20 mM sodium acetate, 2 M NaCl, pH 4.5, 20 column volumes). The main elution peak was pooled, concentrated, and buffer exchanged into D-PBS at least three times. The eluted IL-2 and IL-2 variants were further purified on a Superdex 200 increase column *(GE Healthcare*), with 20 mM Tris-HCl and 500 mM NaCl, pH 8.0, serving as the running buffer. The main elution peak was collected, concentrated, and frozen at −80 °C until use. For WT-IL-2, the yield was approximately 30 mg/L after three rounds of purification. For FSY-bearing IL-2 variants, the yield was approximately 1 to 5 mg/L after three rounds of purification depending on the site of FSY incorporation. The purities of proteins obtained via this method typically exceeded 95%as determined by Coomassie Blue analysis.

**Mass spectrometry analysis**

Site-specific FSY incorporation was confirmed by peptide mapping as previously reported^2,3^. Following trypsin digestion of the purified FSY-bearing IL-2 overnight at 37 °C, the reaction was quenched by the addition of trifluoroacetic acid (TFA). The tryptic peptides were resuspended and separated by HPLC on a C18 RP column. The fractions were collected, dried, and resuspended in TFA, after which the peptides were identified by MALDI-TOF-TOF analysis. To validate the proximity-enabled SuFEx reaction upon protein binding, the IL-2-FSY/IL-2Rα-crosslinked protein samples were digested with trypsin, and the digested peptides were analysed with an in-line EASY-spray source and a nano-LC UltiMate 3000 high-performance liquid chromatography system interfaced with a Q Exactive HF mass spectrometer as previously reported^1^. The tryptic peptides were separated on aC18 RP-HPLC column and detected by a Q Exactive HF mass spectrometer followed by twenty high energy collisional dissociation (HCD) MS/MS scans. A dynamic exclusion time of 30 s was used, and singly charged ions were excluded. Raw MS data were searched by pLink.

**Binding affinity analysis**

The binding affinities of IL-2 and IL-2 (FSY) towards IL-2Rα were measured by BLI using an Octet RED96 instrument (*Forté Bio, Pall Life Sciences*). All assays were performed in D-PBS supplemented with 0.2% BSA and 0.05% Tween 20 (assay buffer). In total, 200 μl of each solution was added per well. Assays were performed at 25 °C in solid black 96-well plates (Geiger Bio-One). His-tagged IL-2Rα in capture buffer (20 μg/ml, acetic acid-sodium acetate, pH 4.0) was chemically immobilized by amine coupling on an AR2G biosensor according to the manufacturer’s directions. Two-fold dilution series (1.5-100 nM) of IL-2 and L72-FSY were used in a titration series of seven. The dissociation of the interaction was followed for 300 s. No regeneration was needed between measurements because the dissociation was completed within 10 min. The data were analysed using Forté Bio data acquisition software v.8.1 and fitted using a global fit 1:1 model to determine the K_D_ values and other kinetic parameters.

**Pharmacokinetic analysis in mice**

The PK properties of WT-IL-2, L72-FSY and their PEGylated analogues were determined following their administration to groups of female C57BL/6 and B-hIL2RA mice (3 mice per group, 8 to 10 weeks old) that received single subcutaneous injections at a dose of 5 µg per mouse. The dose concentrations reflected the weight of the IL-2 protein without the attached PEG. Mice were bled via the retro-orbital method at various time points (30 min and 2, 8, 24, 48, 72 and 96 hours post injection). The blood samples were centrifuged, and the plasma samples were stored at -80 °C until analysis. A predose sample (0 hours) was drawn 1 day prior to injection of the test samples. The levels of the assayed proteins were quantified using a commercial Sandwich ELISA Kit (*Alpha Diagnostic International, USA*). PK parameters were estimated using the modelling programme Kinetica (version 5.1; *Thermo, USA*). The NCA Assistant extravascular method (one compartment, no lag time, first-order elimination) was used, and concentration data were uniformly weighted.

**Pathological kidney and lung assessment**

For pathological kidney assessment, in brief, one kidney was fixed with buffered 10% formalin and embedded in a paraffin block. Sections were cut at a thickness of approximately 5 µm and then stained with haematoxylin and eosin (H&E) and periodic acid-Schiff (PAS). The sections were graded by a single renal pathologist who was blinded to the treatment modality for glomerular, interstitial and vascular lesions according to a semiquantitative grading scheme as previously reported^4^. For the determination of IgG and C3 deposition, the other kidney was snap frozen and cut into 5-µm-thick sections. Kidney cryosections were stained with anti-mouse IgG-FITC or IgM-Alexa Fluor 647for the determination of antibody deposition and stained with a rat anti-C3 antibody followed by goat anti-mouse-IgG-H&L-Alexa Fluor 488 (*Abcam, USA*) for the determination of C3 deposition. The slides were visualized by confocal microscopy, and the deposition of the above markers was quantified based on the MFI using ImageJ (version 1.52t). For pathological lung assessment, parts of the lungs were fixed with 10% formalin, embedded in paraffin, sectioned, and stained with H&E. The sections were semiquantitatively scored by a single pathologist blinded to the treatment modality, and inflammation scores were estimated from 20 randomly selected fields of the right and left lungs based on the following symptoms: 0, no inflammation; 1, mild, inflammatory cell infiltration of the perivascular and peribronchiolar space; 2, moderate, inflammatory cell infiltration of the perivascular and peribronchiolar space with modest extension into the lung parenchyma; and 3, severe, inflammatory cell infiltration of the perivascular and peribronchiolar space and large inflammatory foci in the lung parenchyma^5^. The Animal Use Protocol was reviewed and approved annually by the Ethics Review Board of PUMC Hospital, Chinese Academy of Medical Science (CAMS).

**References**

1. Wang, N.*, et al.* Genetically Encoding Fluorosulfate-l-tyrosine To React with Lysine, Histidine, and Tyrosine via SuFEx in Proteins in Vivo. *J Am Chem Soc* **140**, 4995-4999 (2018).

2. Li, Q.*, et al.* Developing Covalent Protein Drugs via Proximity-Enabled Reactive Therapeutics. *Cell* **182**, 85-97 e16 (2020).

3. Zhang, B.*, et al.* Development of next generation of therapeutic IFN-alpha2b via genetic code expansion. *Acta Biomater* **19**, 100-111 (2015).

4. Tao, X., Fan, F., Hoffmann, V., Longo, N.S. & Lipsky, P.E. Therapeutic impact of the ethyl acetate extract of Tripterygium wilfordii Hook F on nephritis in NZB/W F1 mice. *Arthritis Res Ther* **8**, R24 (2006).

5. Wang, J.*, et al.* Klebsiella pneumoniae alleviates influenza-induced acute lung injury via limiting NK cell expansion. *J Immunol* **193**, 1133-1141 (2014).


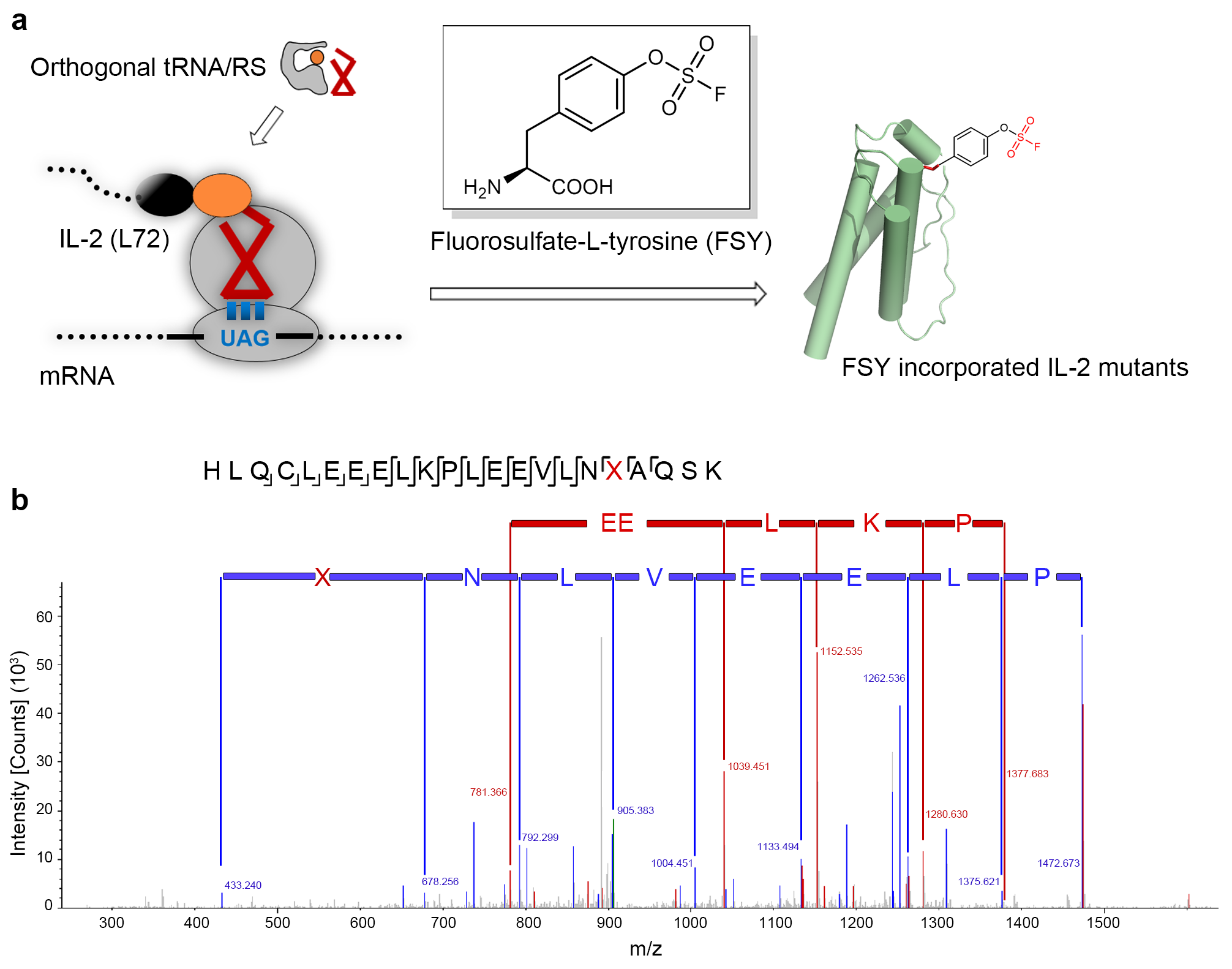


**Supplementary Figure 1** Amber codon suppression-mediated site-specific incorporation of fluorosulfate-L-tyrosine (FSY) into human IL-2. **a** Schematic representation of the amber codon suppression-mediated incorporation of FSY into IL-2 at the desired positions. Briefly, an orthogonal amber suppressor tRNA consisting of an aminoacyl-tRNA synthetase (aaRS) pair was used to site-specifically incorporate an unnatural amino acid (FSY) in response to an amber nonsense codon. The fluorosulfate group of FSY remains inert inside the protein and is able to react with Try, His, and Lys residues in proximity via a click chemistry sulfur-fluoride exchange, forming a stable linkage resistant to hydrolysis. See also Fig. 1a. **b** Tandem mass spectrum of L72-FSY. The representative partial sequence of the peptide containing FSY, HLQCLEEELKPLEEVLNXAQSK, can be read from the annotated b (red) or y (blue) ion series. A series of partial sequences containing L72FSY can be read from the annotated ion series. X represents FSY.


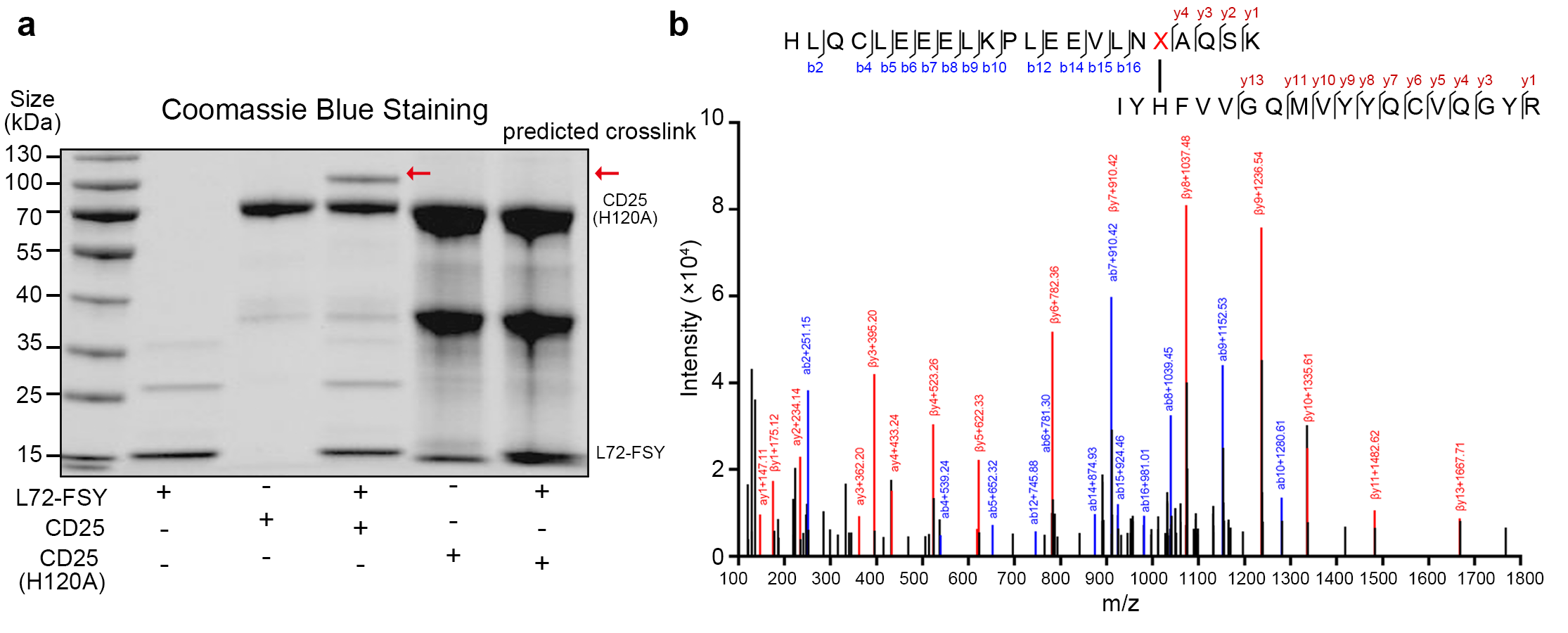


**Supplementary Figure 2** Validation of the covalent crosslinking between L72-FSY and H120 of IL-2Rα. **a** The mutation of His120 to Ala in IL-2α abolished its covalent binding to L72-FSY. The mutated IL-2Rα (H120A) with human IgG1-Fc-tag was expressed using the mammalian Freestyle 293 system and purified by protein A/G. Validation of the covalent crosslinking was similarly performed as Fig 1c. **b** Validation of the covalent crosslinking between L72-FSY and H120 of IL-2Rα using tandem mass spectrometry. The representative cross-linked sequence of the peptide containing FSY of IL-2 and H120 of IL-2Rα can be read from the annotated ion series. X represents FSY. See also Fig 1.


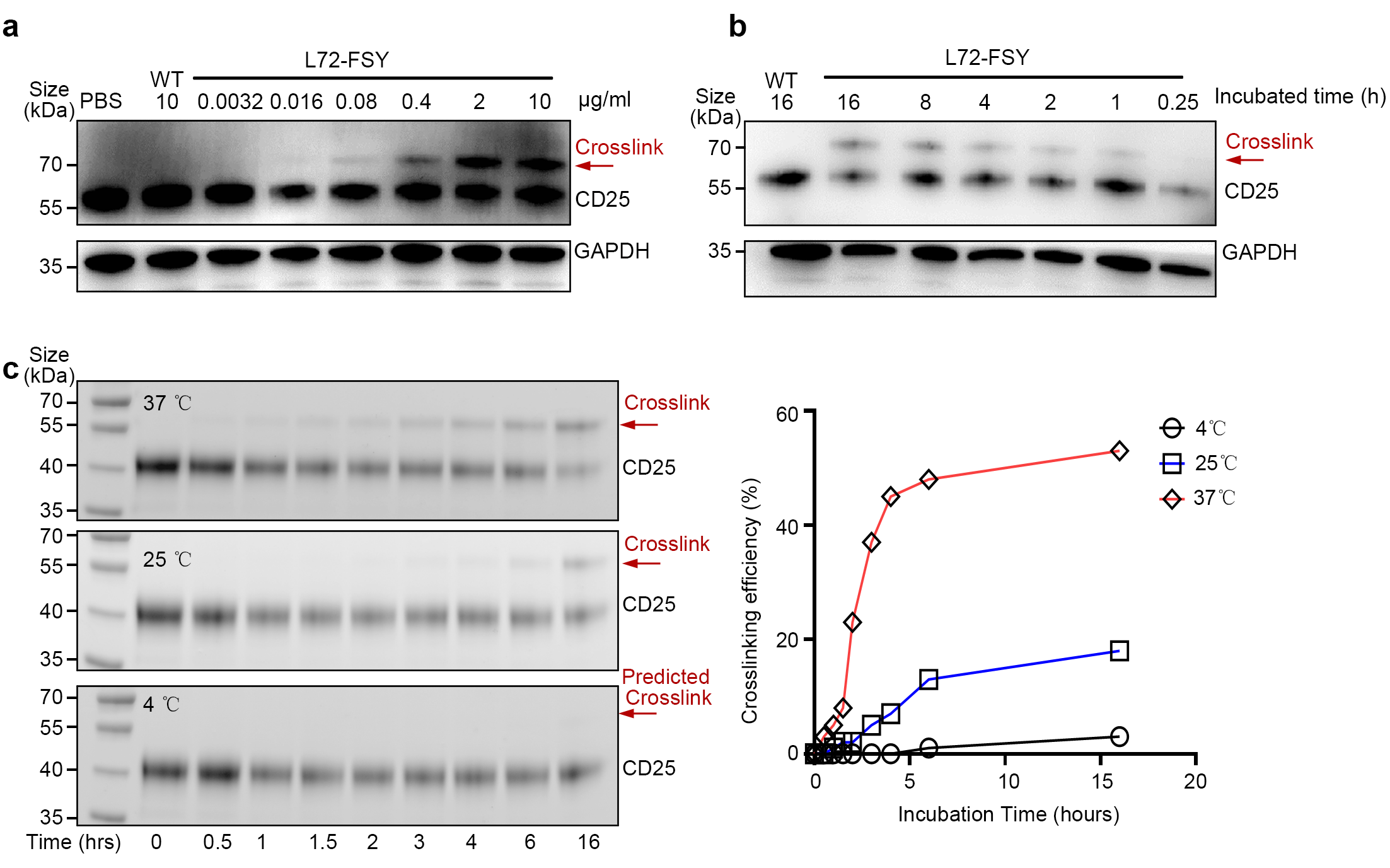


**Supplementary Figure 3** Characterization of the covalent binding of L72-FSY in vitro. **(a-b)** Characterization of the covalent binding of L72-FSY on IL-2α expressing YT cells (CD25-YT). YT cells expressing full-length IL-2Rα were incubated at 37 ℃ in the presence of the indicated amount of L72-FSY for the indicated amounts of time, and the denatured supernatant of the cell lysate was analysed by WB with an anti-IL-2Rα primary antibody. **a** L72-FSY covalently bound to IL-2Rα on the surface of CD25-YT cells in a dose-dependent manner, and (**b**) this binding was obvious within 1 hour under physiological conditions (2 ug/ml, 37 °C, pH = 7.4). **c** L72-FSY covalently binds to IL-2Rα in a time- and temperature-dependent manner in vitro as determined by molecular analysis. SDS-PAGE and Coomassie Blue analyses of L72-FSY (10 μM) crosslinking to the extracellular domains of IL-2Rα (IL-2Rα-His) at the indicated time points and temperatures (left). The crosslinking efficiencies of the indicated treatments were evaluated based on densitometry detection using ImageJ (right). Representative results from one of two experiments are shown.


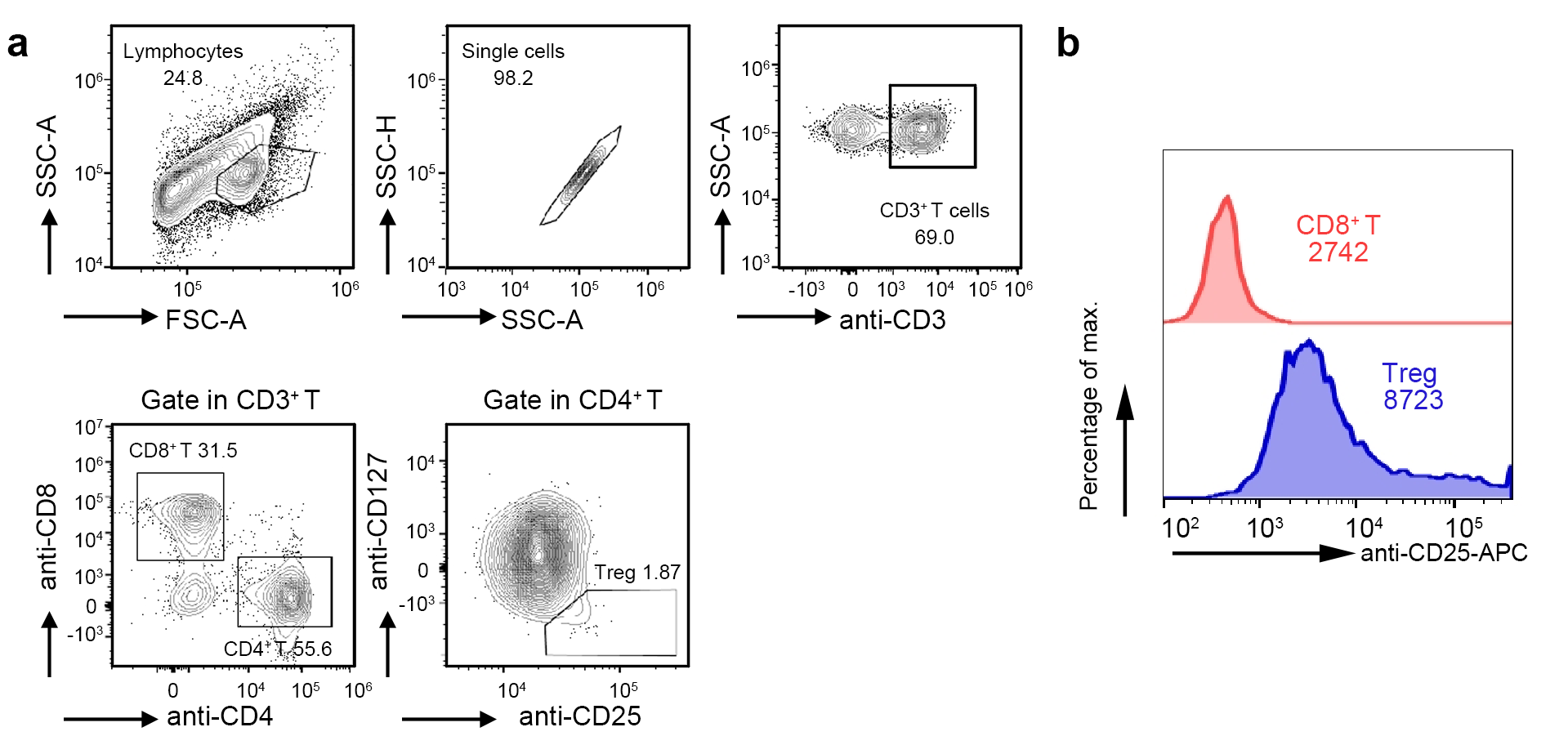


**Supplementary Figure 4** Validation of CD25 expression on Treg cells and CD8^+^ T cells used in the in vitro assay. **a** Representative flow plots of the gating strategy. Treg cells are defined as CD3^+^CD8^-^CD4^+^CD25^high^CD127^low^, and CD8^+^T cells are defined as CD3^+^CD4^-^CD8^+^T cells. **b** The MFIs of CD25 on Treg and total CD8^+^ T cells were determined. Representative results from one of at least three experiments are shown. Treg cells were isolated and expanded ex vivo for 9 days and then allowed to rest for 1 day prior to the assay. CD8^+^ T cells were isolated and analysed immediately.


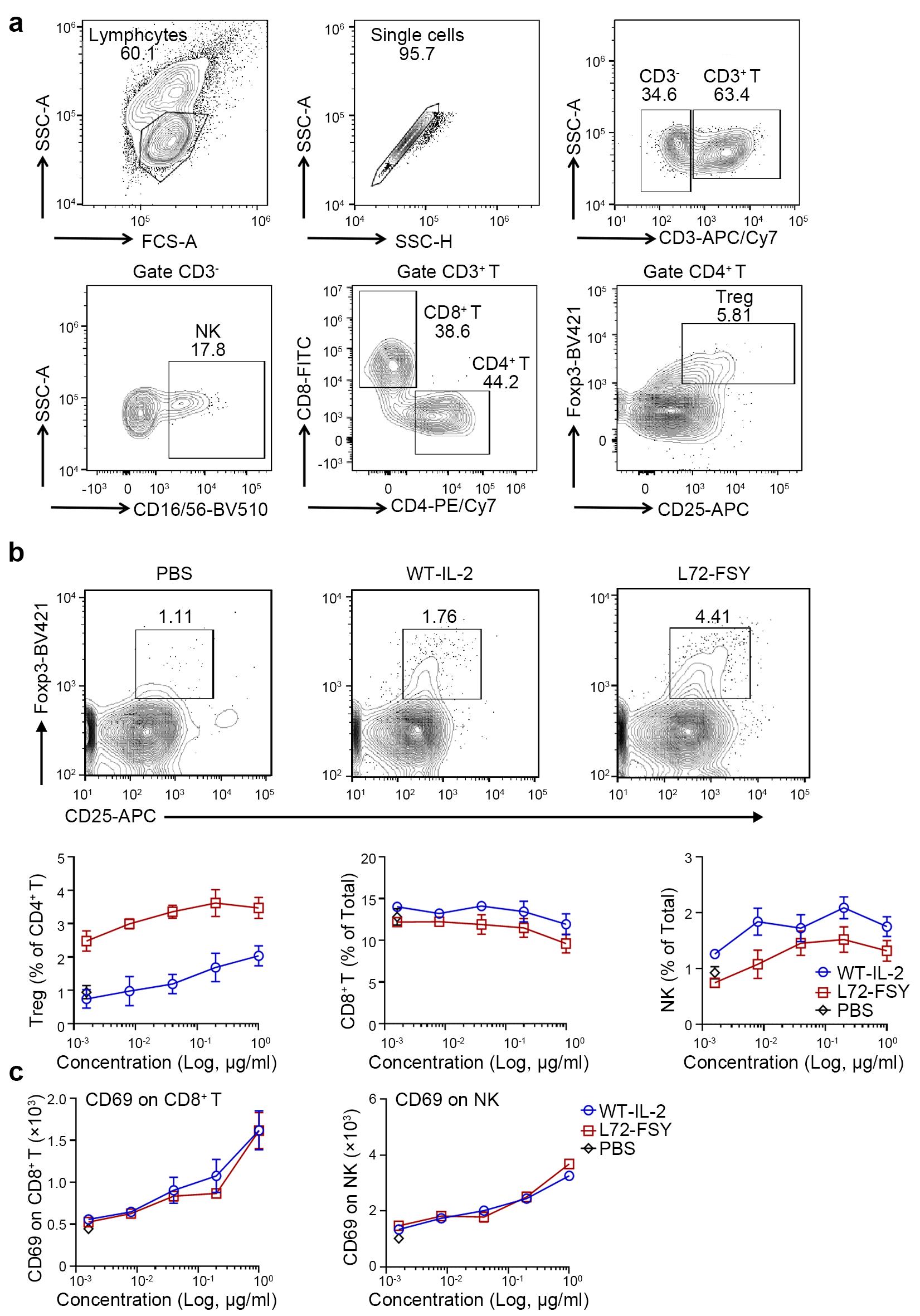


**Supplementary Figure 5** L72-FSY preferentially promotes Treg cells *in vitro*. **a** The gating strategies of Treg (CD3^+^CD4^+^CD25^high^Foxp3^+^), CD8^+^T (CD3^+^CD4^-^CD8^+^) and NK (CD3^-^CD16^+^56^+^) cells used in assessment. **b** Dose responses of the proportions of Tregs, CD8^+^ T or NK cells after the indicated treatment. Representative flow cytometry histograms showing the percentages of Tregs among the total CD4^+^ T cells in response to incubation with 0.2 µg/ml WT-IL-2 or L72-FSY. **c** The expression of CD69 on CD8^+^ T and NK cells in the presence of the indicated concentrations of the indicated samples was evaluated. For panel **b** and **c**, hPBMCs were incubated with the samples at the indicated concentrations for three days and then stained for flow cytometry analysis. The symbol is plotted as the mean of triplicate wells, with the error bars representing the SEM (n=3), and one representative donor of three is shown. See also Fig. 3a.

**
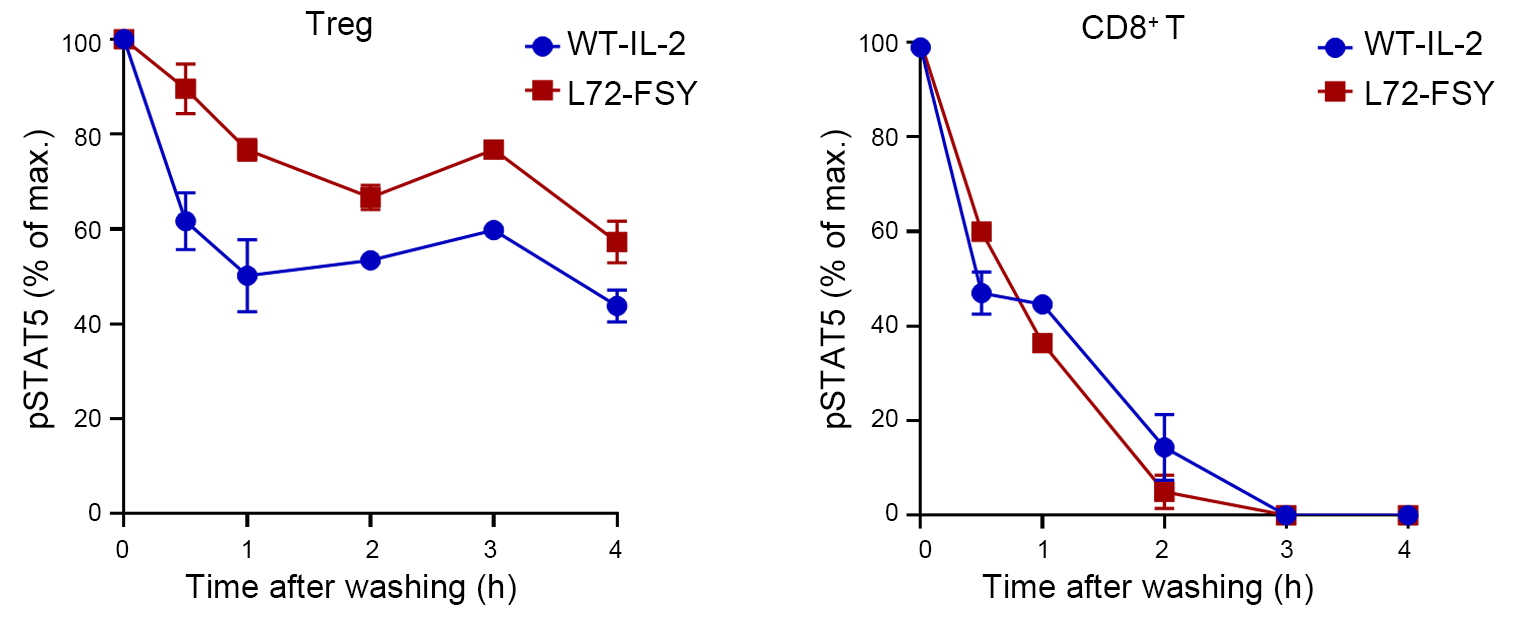
**

**Supplementary Figure 6** L72-FSY selectively resulted in sustained pSTAT5 signalling in Tregs. Tregs or CD8^+^ T cells were pulsed with L72-FSY or WT-IL-2 at 37 °C for 1.5 hours and were then acid-washed. The cells were then recultured at 37 °C and assayed for pSTAT5 at the indicated time points. The symbol is plotted as the mean of triplicate wells, with the error bars representing the SEM derived from one donor, and the data are representative of three individual donors (n=3).

**
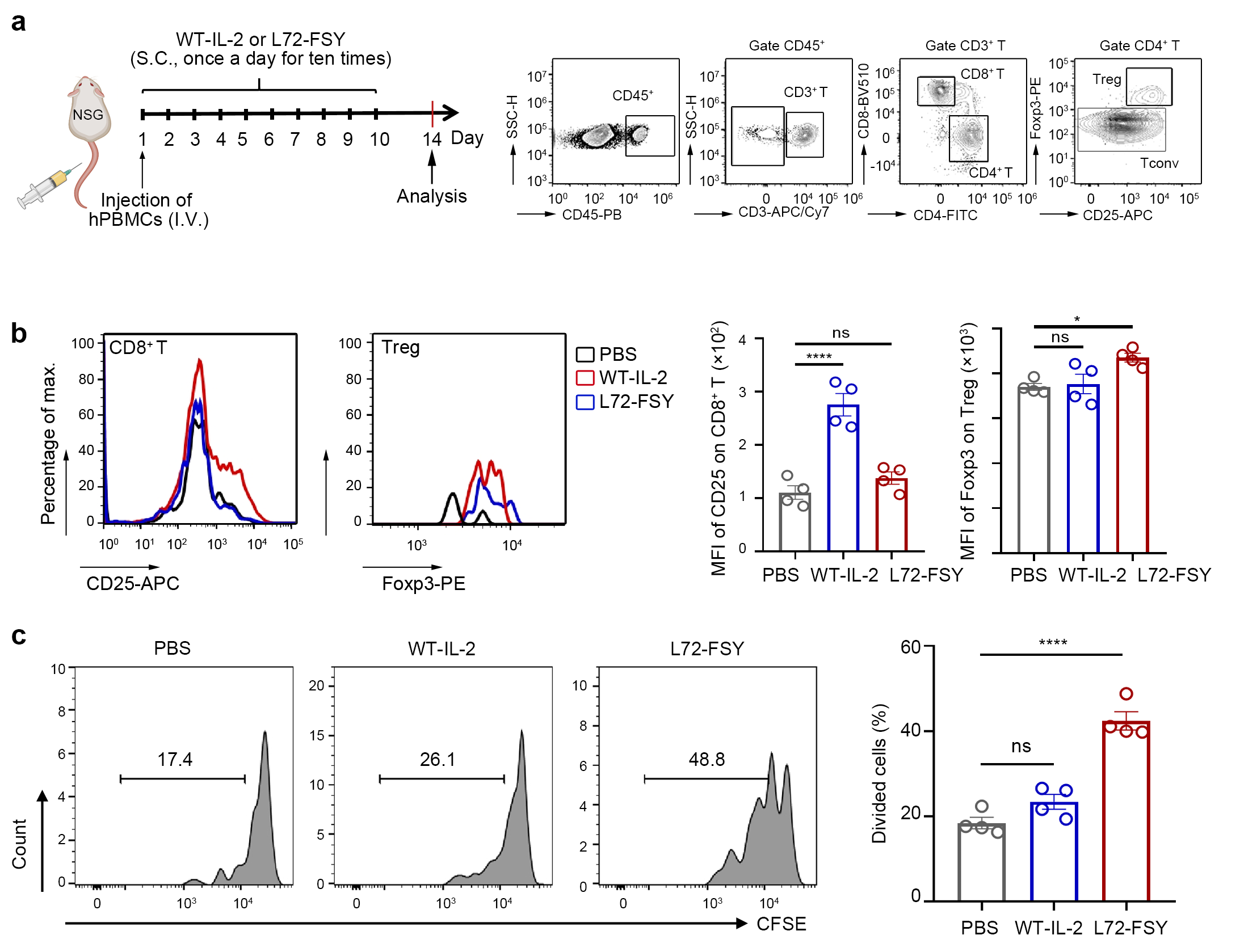
**

**Supplementary Figure 7** L72-FSY preferentially promoted Tregs in a humanized NSG model in vivo. **a** The scheme and gating strategy for the experiment are shown. NSG mice were injected with hPBMCs, followed by the subcutaneous administration of 2 μg of WT-IL-2 or L72-FSY daily for ten consecutive days. Splenocytes were harvested five days after the last injection for flow cytometry analysis. **b** Flow cytometry histograms and MFI bar graphs of induced CD25 and Foxp3 expression on [splenic](javascript:;) CD8^+^ T cells or Tregs in response to the indicated treatments. See also Fig. 3c-d. **c** L72-FSY selectively potentiated the growth of adoptively transferred Tregs in the recipient mouse. NSG mice were cotransferred with isolated human CD4^+^ Tconv cells and Tregs at a ratio of 1:1, followed by the administration of 2 μg of WT-IL-2 or L72-FSY daily for five consecutive days. The Tregs were expanded for two weeks following isolation and labelled with CFSE prior to injection. Representative flow cytometry histograms measuring transferred Treg proliferation. The bar graph shows the percentage of dividing Tregs in response to the indicated treatment. See also Fig 3e. Data are representative of at least 4 (panel **a-b**) or 2 (panel **c**) independent experiments and are presented as the mean ± SEM of four mice per group (n=4). The p values were determined by one-way ANOVA (Dunnett’s multiple-comparison test compared with the PBS control), *p ≤ 0.05, ****p ≤ 0.0001.

**
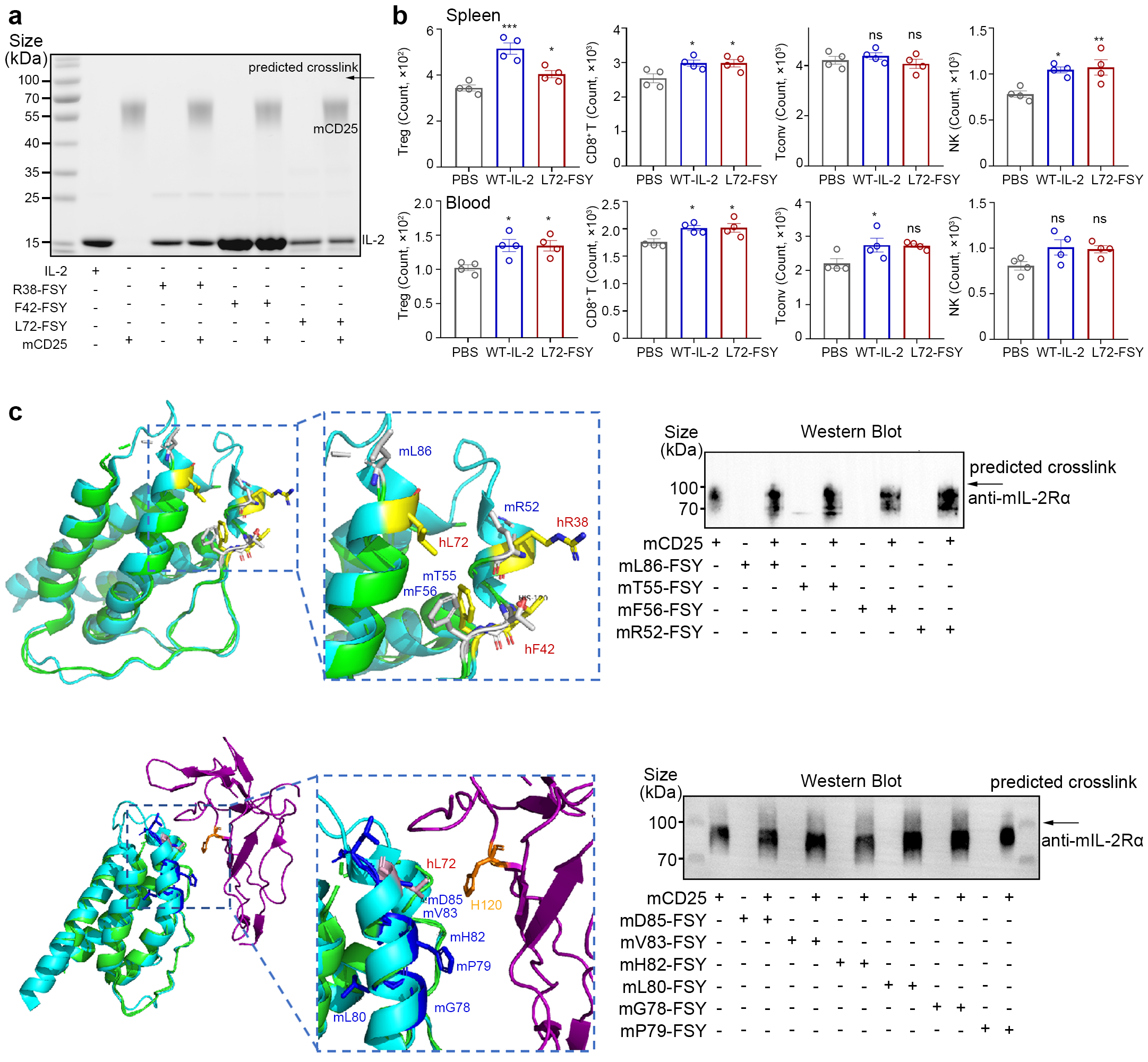
**

**Supplementary Figure 8** Validation of the specificity of covalently bound IL-2 *in vitro* and *in vivo*. **a** FSY-bearing human IL-2 variants did not covalently crosslink to mouse IL-2Rα. Equal doses of purified WT- and FSY-bearing human IL-2 were incubated with the ectodomain of mouse IL-2Rα (mIL-2Rα) at room temperature overnight, and the denatured samples were then stained with Coomassie blue. The arrow indicates the expected covalent crosslinking of human IL-2 and mIL-2Rα based on molecular weight. **b** Validation of the effects of L72-FSY in health C57BL/6 mice in vivo. C57BL/6 mice were subcutaneously injected with PBS, WT-IL-2 (human) or L72-FSY (2 μg) daily for ten consecutive days. Blood and splenocytes were harvested five days after the last injection for flow cytometry analysis. Bar graphs show the indicated cell count after the indicated treatment. The data are presented as the mean ± SEM of four mice per group (n=4). The p values were determined by one-way ANOVA (Dunnett’s multiple-comparison test compared with the PBS control), *p ≤ 0.05, **p ≤ 0.01, ***p ≤ 0.001. Representative results from one of two experiments are shown. **c** Analysis of the covalent binding of FSY-bearing mouse IL-2 (mIL-2) variants to mIL-2Rα. A series of sites within mouse IL-2 that potentially bind to IL-2Rα were selected based on the alignment of human and mouse IL-2 (PDB numbers 2ERJ and 4YQX for human and mouse IL-2, respectively). Equal doses of the resultant mIL-2 variants containing FSY were incubated with Fc-tagged ectodomain of mIL-2Rα (mIL-2Rα-Fc) at room temperature overnight, and the denatured samples were then analysed by WB. The anti-mouse CD25 antibody was used as the primary WB antibody. The arrow indicates the expected covalent crosslinking of mIL-2 and mIL-2Rα based on molecular weight.

**
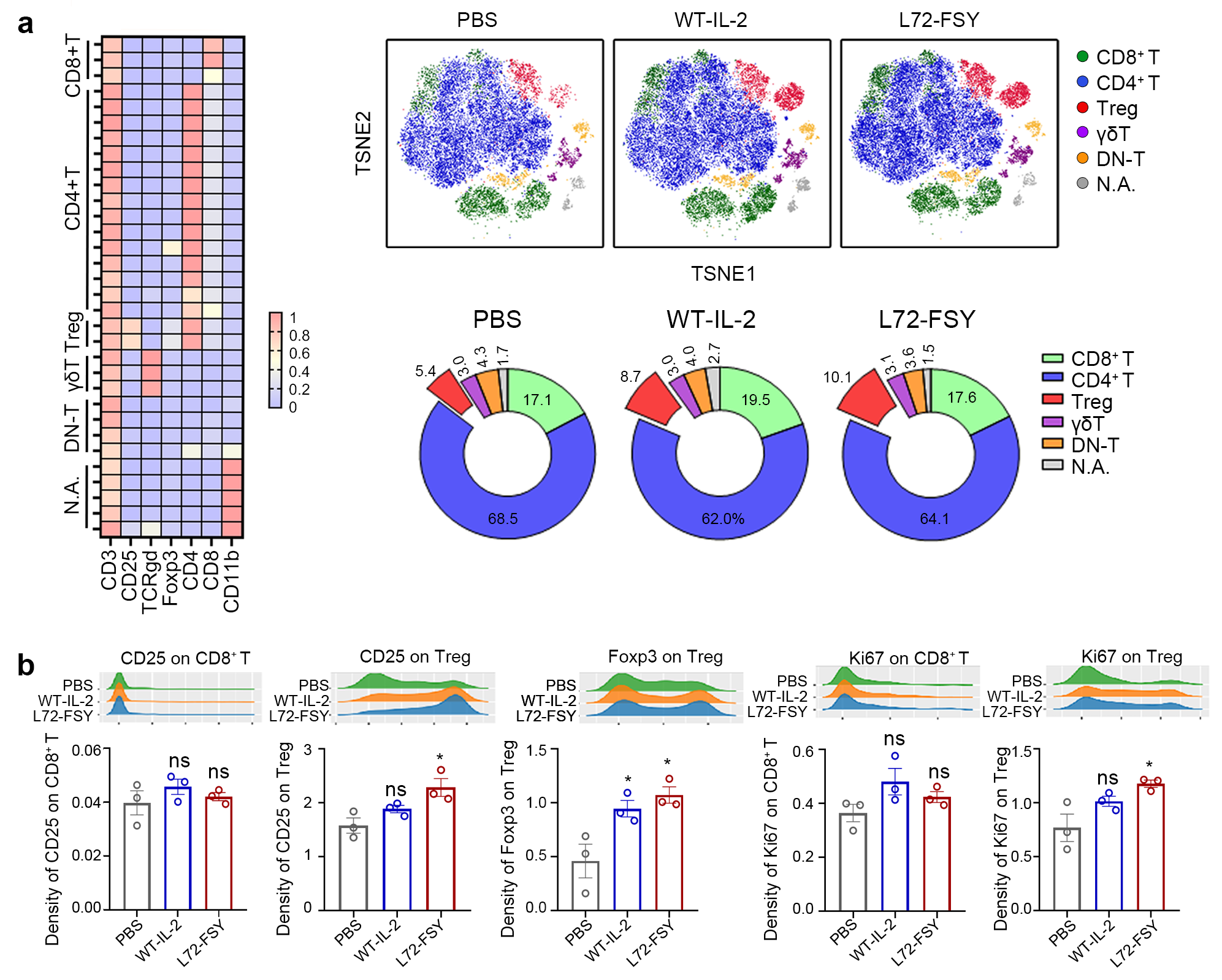
**

**Supplementary Figure 9** Validation of the Treg-selective effects of L72-FSY in B-hIL2RA mice by mass cytometry. **a** Profiling of splenic T cell clusters in response to the indicated treatment. CD25-humanized B6 mice (B-hIL2RA mice, three mice per group, n=3) were subcutaneously injected with PBS, WT-IL-2 or L72-FSY (2 μg) daily for ten consecutive days, and splenocytes were harvested five days after the last injection for mass cytometry. Mice treated with PBS were used as control. The heat map representing the expression of the indicated markers within the five cellular clusters was used to annotate clusters (left). The tSNE visualization of the FlowSOM-generated T cell cluster (CD3^+^CD19^-^) and pie chart displaying the frequencies of immune T cell subsets in response to the indicated treatment were shown. **b** Examination of functional and proliferative biomarkers in the indicated cells after the indicated treatment by mass cytometry. Bar graphs and representative flow cytometry histograms show densities of CD25, Foxp3, and Ki67 on the indicated cells after the indicated treatment. The data are presented as the mean ± SEM of three mice per group (n=3). The p values were determined by one-way ANOVA (Dunnett’s multiple-comparison test compared with PBS group), *p ≤ 0.05. See also Fig. 4a.

**
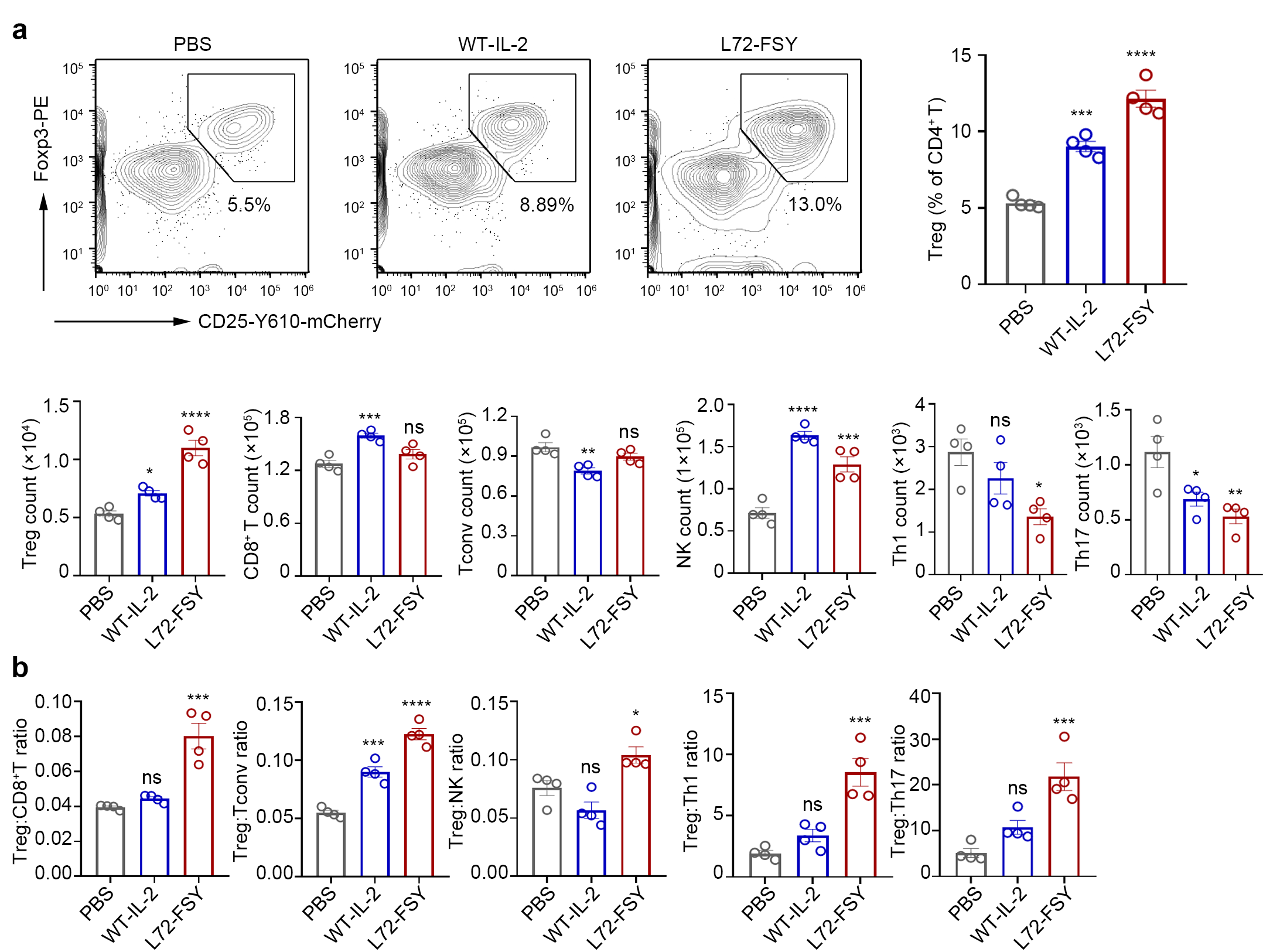
**

**Supplementary Figure 10** L72-FSY selectively expanded mouse Tregs in B-hIL2RA mice *in vivo*. B-hIL2RA mice were subcutaneously injected with WT-IL-2 or L72-FSY (2 μg) daily for ten consecutive days, and PBS was applied as a control. Splenocytes were collected five days after the last injection for flow cytometry analysis. **a** Profiling of various types of immune cell populations, including the percentage of Tregs among CD4^+^ T cells and total counts of Treg, CD8^+^ T, Tconv, NK, Th1 and Th17 cells, in the spleen of mice with the indicated treatment on day 5 after the last injection. **b** The resultant ratios of Treg:CD8^+^T, Treg:Tconv, Treg:NK, Treg:Th1, and Treg:Th17 cells in response to the indicated treatment. The data are presented as the mean ± SEM of four mice per group (n=4). The p values were determined by one-way ANOVA (Dunnett’s multiple-comparison test compared with PBS group), *p ≤ 0.05, **p ≤ 0.01, ***p ≤ 0.001, ****p≤ 0.0001. Data are representative of three independent experiments.


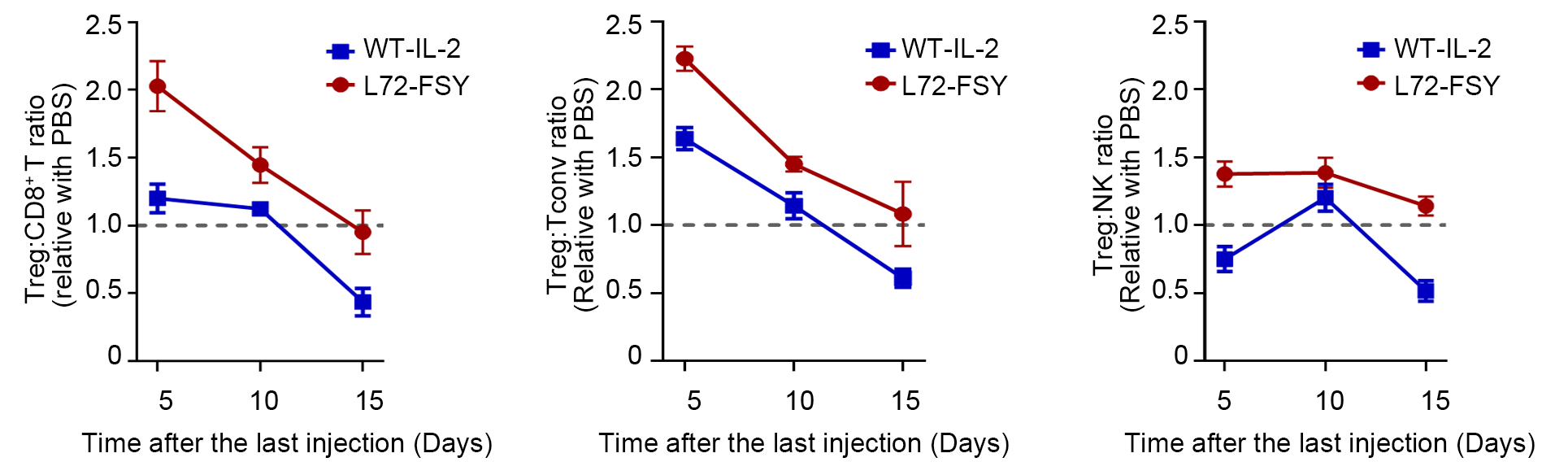


**Supplementary Figure 11** The time curves of the relative ratios of Treg to CD8^+^ T, Tconv, and NK cells at the indicated time points after the indicated treatment. B-hIL2RA mice were subcutaneously injected with WT-IL-2 or L72-FSY (2 μg) daily for ten consecutive days, and PBS was applied as a control. Splenocytes were collected at the indicated time point for flow cytometry analysis. The data are shown as the value relative to that of PBS-treated mice and presented as the mean ± SEM of four mice per group (n=4). Data are representative of two independent experiments.


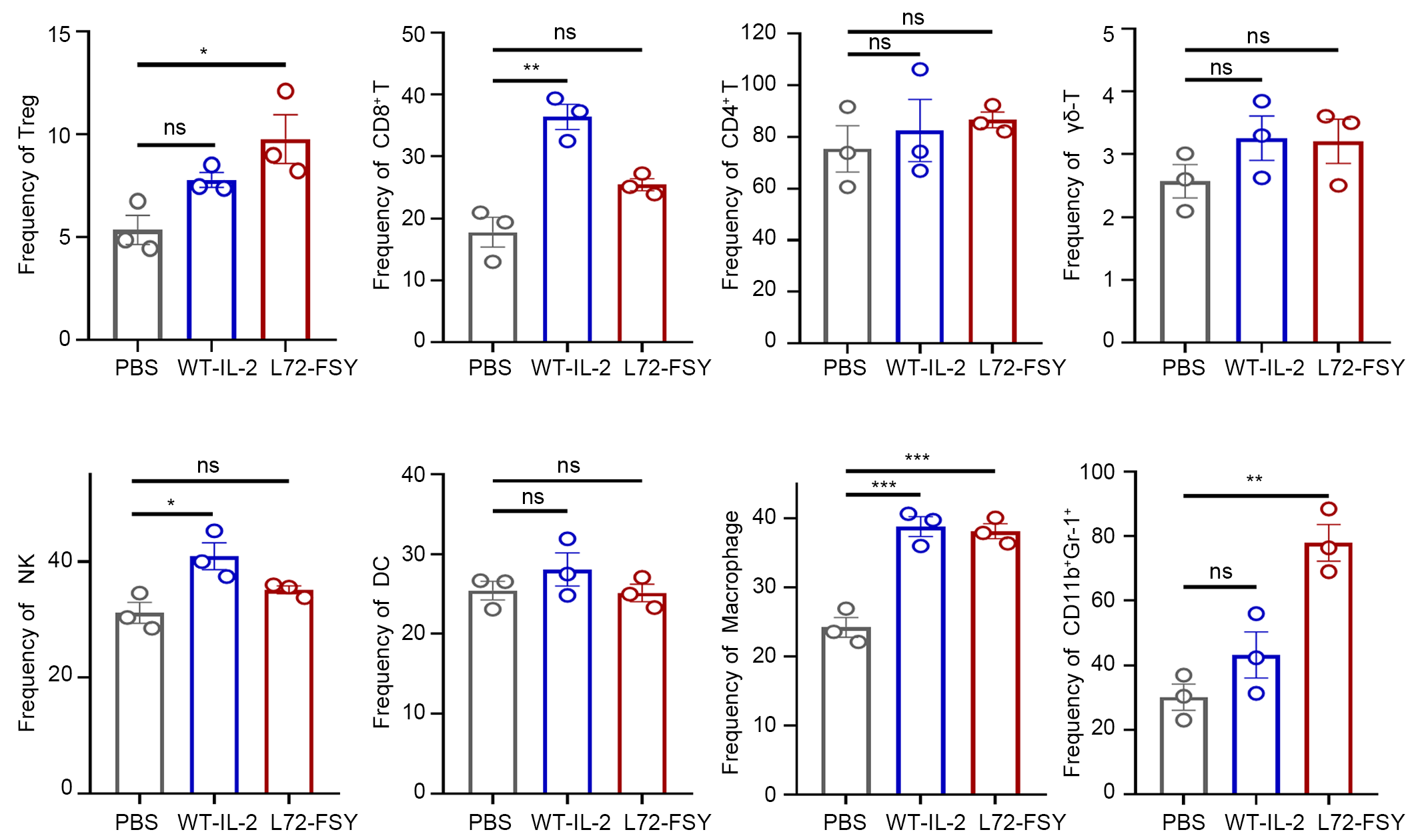


**Supplementary Figure 12** Comparison of cluster frequencies in response to indicated treatment in B-hIL2RA mice by mass cytometry. Bar graphs show the indicated cluster frequency after the indicated treatment. The data are presented as the mean ± SEM of three mice per group (n=3). The p values were determined by one-way ANOVA (Dunnett’s multiple-comparison test compared with PBS group), *p ≤ 0.05, **p ≤ 0.01, ***p ≤ 0.001. See also Fig. 4a and Supplementary Fig. 9.


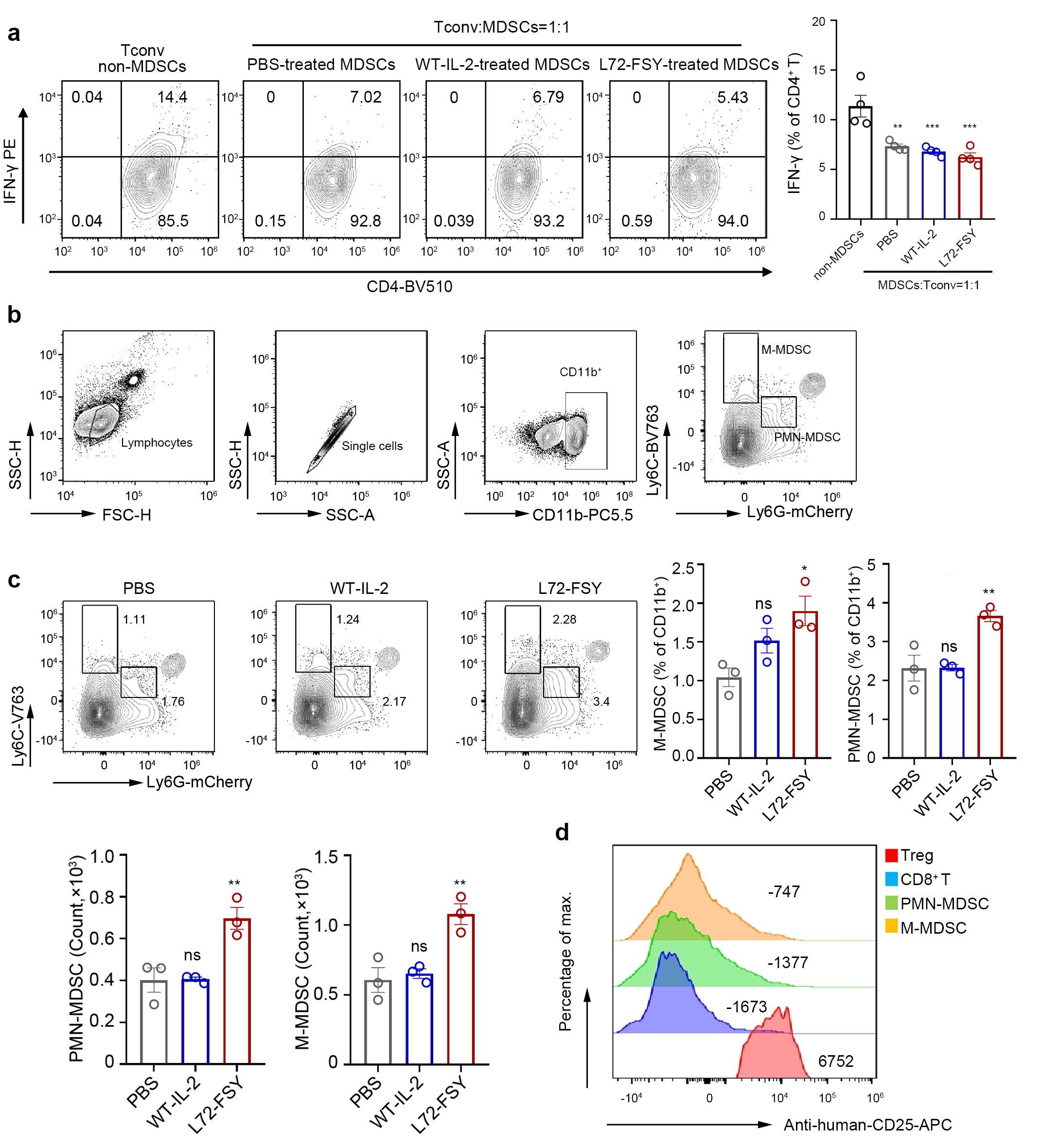


**Supplementary Figure 13** Validation of the MDSC-selective effects of L72-FSY *in vitro* and *in vivo*. **a** Validation of the inhibitory effect of isolated CD11b^+^Gr-1^+^ cells *in vitro*. The isolated CD4^+^ T cells stimulated with anti-CD3/anti-CD28 beads were cultured alone or cocultured with CD11b^+^Gr-1^+^ cells isolated from mice with the indicated treatment at a ratio of 1:1 for 3 days. Representative flow plots and bar graphs reflecting the percentage of IFN-γ^+^ cells among CD4^+^ T cells. The symbol represents the mean of quadruplicate wells derived from four mice, and the error bars represent the SEM (n=4). **(b-d)** Validation of the MDSC-selective effects of L72-FSY in a B-hIL2RA mouse model *in vivo*. B-hIL2RA mice were subcutaneously injected with WT-IL-2 or L72-FSY (2 μg) daily for ten consecutive days, and PBS was applied as a control. Splenocytes were collected five days after the last injection for flow cytometry analysis. **b** Gating strategy to sort polymorphonuclear- (PMN, CD11b^+^Ly6G^+^Ly6C^low^) and monocytic- (M, CD11b^+^Ly6G^-^Ly6C^high^) MDSCs from splenocytes of treated mice. **c** Representative flow plots showing the percentage of PMN- and M-MDSCs among CD11b^+^ cells, and bar graphs showing the proportions and counts of MDSCs from the indicated treated mice. The data are presented as the mean ± SEM of three mice per group (n=3). **d** Validation of CD25 expression in the indicated cell populations. The MFIs of CD25 on Tregs, total CD8^+^ T cells, PMN-MDSCs, and M-MDSCs from splenocytes were determined by flow cytometry analysis. The p values were determined by one-way ANOVA (Dunnett’s multiple-comparison test compared with non-MDSCs group for panel **a** and PBS group for panels **c**), *p ≤ 0.05, **p ≤ 0.01, ***p ≤ 0.001. Data are representative of two independent experiments for panels **b-d**.


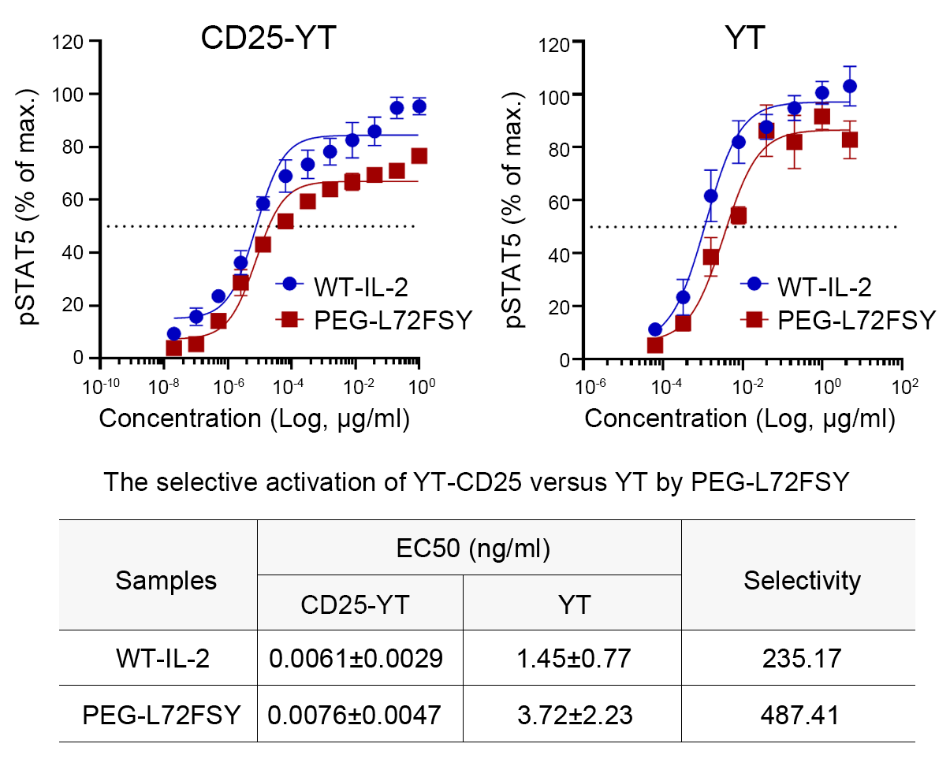


**Supplementary Figure 14** Comparison of the bioactivities and selectivities of PEG-L72FSY and WT-IL-2 using the YT cell model. Dose-dependent assessment of the abilities of PEG-L72FSY and WT-IL-2 to activate YT cells and CD25-YT cells by the pSTAT5 assay. YT and CD25-YT cells were stimulated with the indicated samples in a series of 5-fold dilutions and then stained for quantitative flow analyses. The EC50s and selectivities of WT-IL-2 and PEG-L72FSY were calculated and are shown. The data are presented as the mean ± SD derived from triplicate samples (n=3), and the results from one of three experiments are shown.


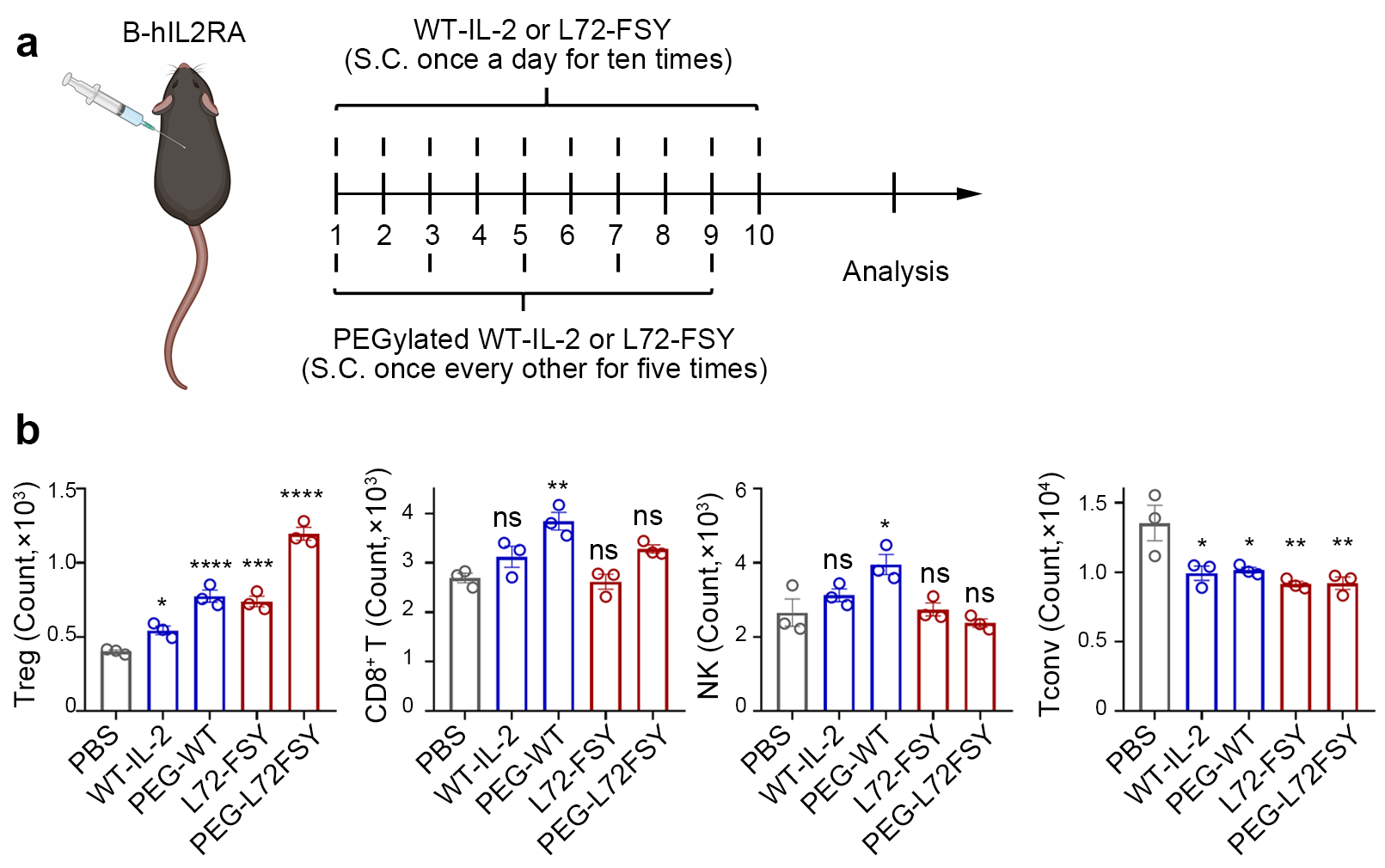


**Supplementary Figure 15** Validation of the Treg-selective effects of PEG-L72FSY on B-hIL2RA mice. **a** Scheme of the experiment. B-hIL2RA mice were subcutaneously injected with 0.5 μg of WT-IL-2 or L72-FSY for ten consecutive days or with an equal dose of their PEGylated forms once every other for a total of five times. Splenocytes were collected five days after the last injection for analysis. **b** Bar graphs showing the numbers of Tregs, CD8^+^ T cells, NK cells and Tconv cells in the indicated mice. The data are presented as the mean ± SEM of three mice per group (n=3), and representative results from one of two experiments are shown. The p values were determined by one-way ANOVA (Dunnett’s multiple-comparison test compared with PBS group), *p ≤ 0.05, **p ≤ 0.01, ***p ≤ 0.001, ****p ≤ 0.0001. See also Fig. 5e.


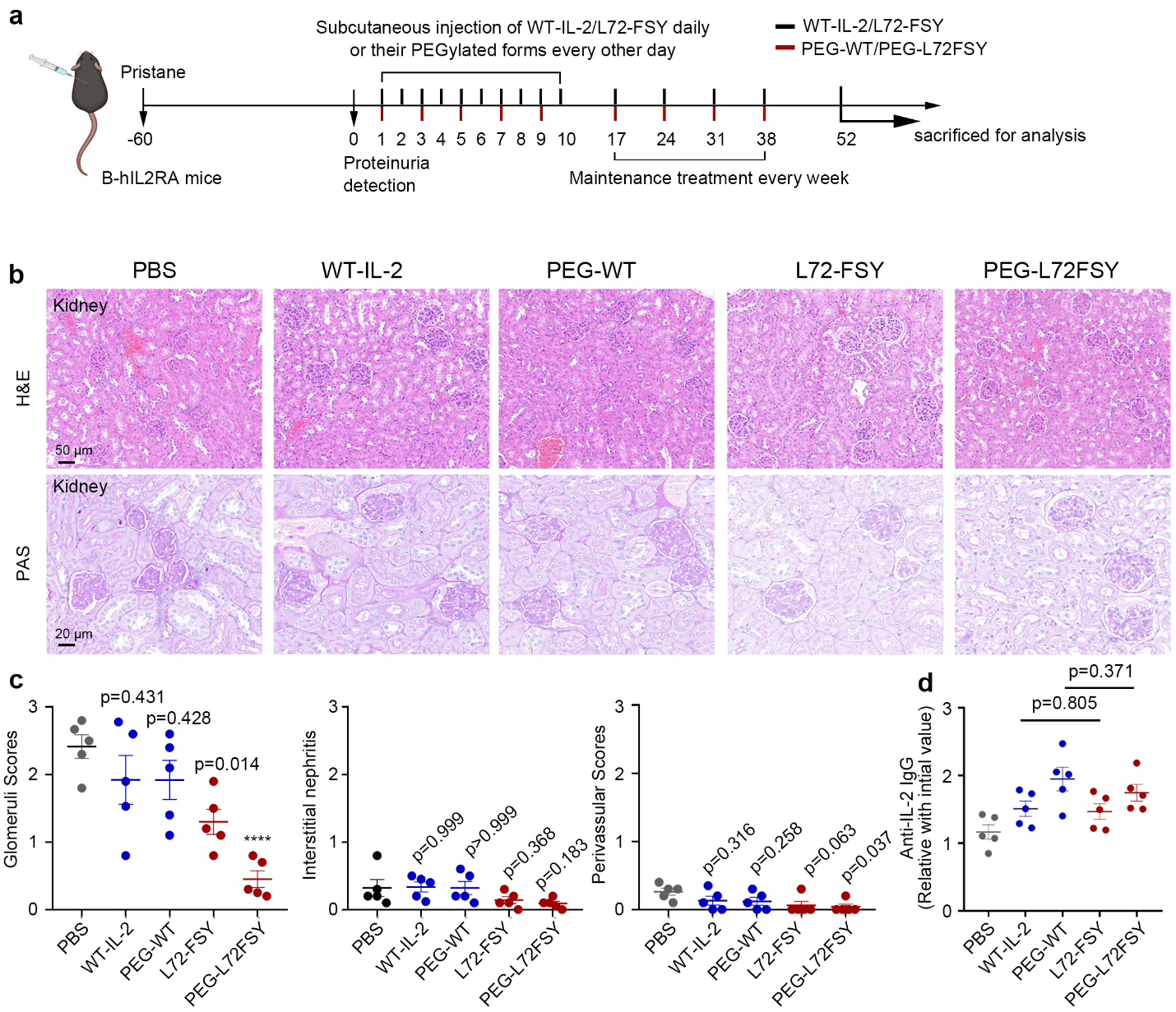


**Supplementary Figure 16** FSY-incorporated IL-2 variants ameliorated kidney without induction of immune response against IL-2 in pristane-treated B-hIL2RA mice. **a** Scheme of the experiment. B-hIL2RA mice were intraperitoneally injected with 0.5 ml of pristane. The establishment of lupus was confirmed by the detection of proteinuria two months later, and subcutaneous injection of the indicated samples at the indicated time points was then performed. The mice were sacrificed two weeks after the final injection for histopathological and immunohistochemical analyses. See also Fig. 6a-b. **b** Haematoxylin and eosin (H&E, top) and periodic acid-Schiff (PAS, bottom) staining of kidney and (**c**) Scores for glomerular, tubulointerstitial, and perivascular lesions according to H&E staining are shown. The p values were determined by one-way ANOVA (Dunnett’s multiple-comparison test compared with PBS group), ****p ≤ 0.0001. **d** Immunogenicity analysis show the titers of antibodies against IL-2 elicited by the indicated treatment at the end of experiment. Unmodified IL-2 without his-tag was immobilized on ELISA plates, and plasma samples were added and incubated. Bound antibodies against IL-2 were detected by secondary incubation with HRP-conjugated anti-mouse IgG antibodies, followed by TMB detection. The p values shown were determined by unpaired two-tailed t-tests. For all panels, the data are presented as the mean ± SEM of five mice per group (n=5), and representative results from one of two experiments are shown.


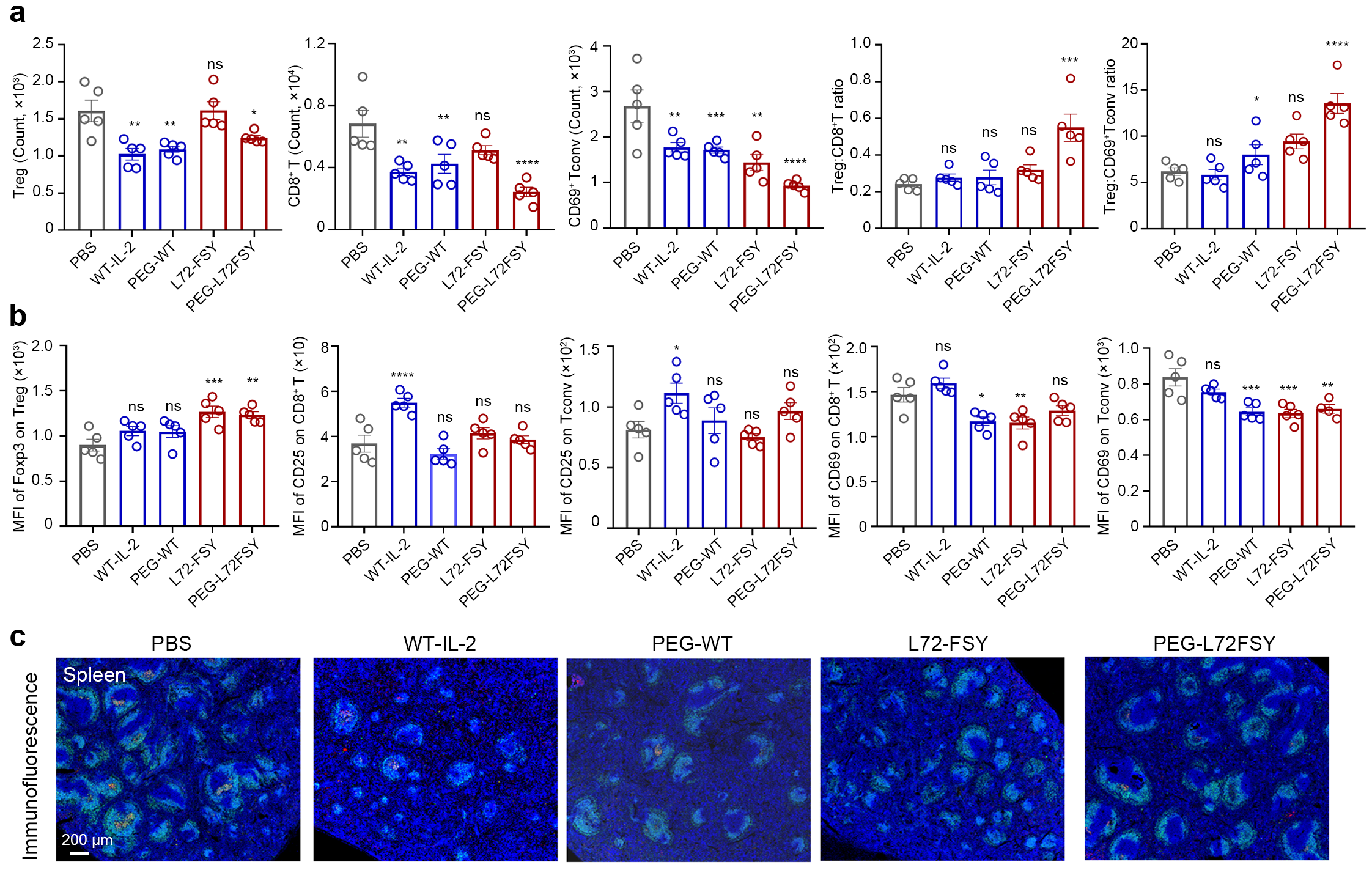


**Supplementary Figure 17** Long-term administration of FSY-incorporated IL-2 variants selectively expanded Tregs in pristane-treated B-hIL2RA mice. **a** Bar graphs showing the counts of Tregs, CD8^+^ T cells, CD69^+^ Tconv cells and the ratio of Tregs/CD8^+^ T cells and Tregs/CD69^+^ Tconv cells in the spleens of mice receiving the indicated treatment. **b** Bar graphs show the MFI of activation markers on the indicated T cells in spleen at the end of experiment. **c** Immunofluorescence examination germinal center (GC) formation in spleen. Cryosections from spleen were stained with anti-B220 (green)/GL7 (red), followed with analysis by fluorescence microscopy. For panel (**a-b**), the data are presented as the mean ± SEM of five mice per group (n=5), the p values shown were determined by one-way ANOVA (Dunnett’s multiple-comparison test compared with PBS treated group). *p ≤ 0.05, **p ≤ 0.01, ***p ≤ 0.001, ****p ≤ 0.0001. See also Fig. 6a-b.


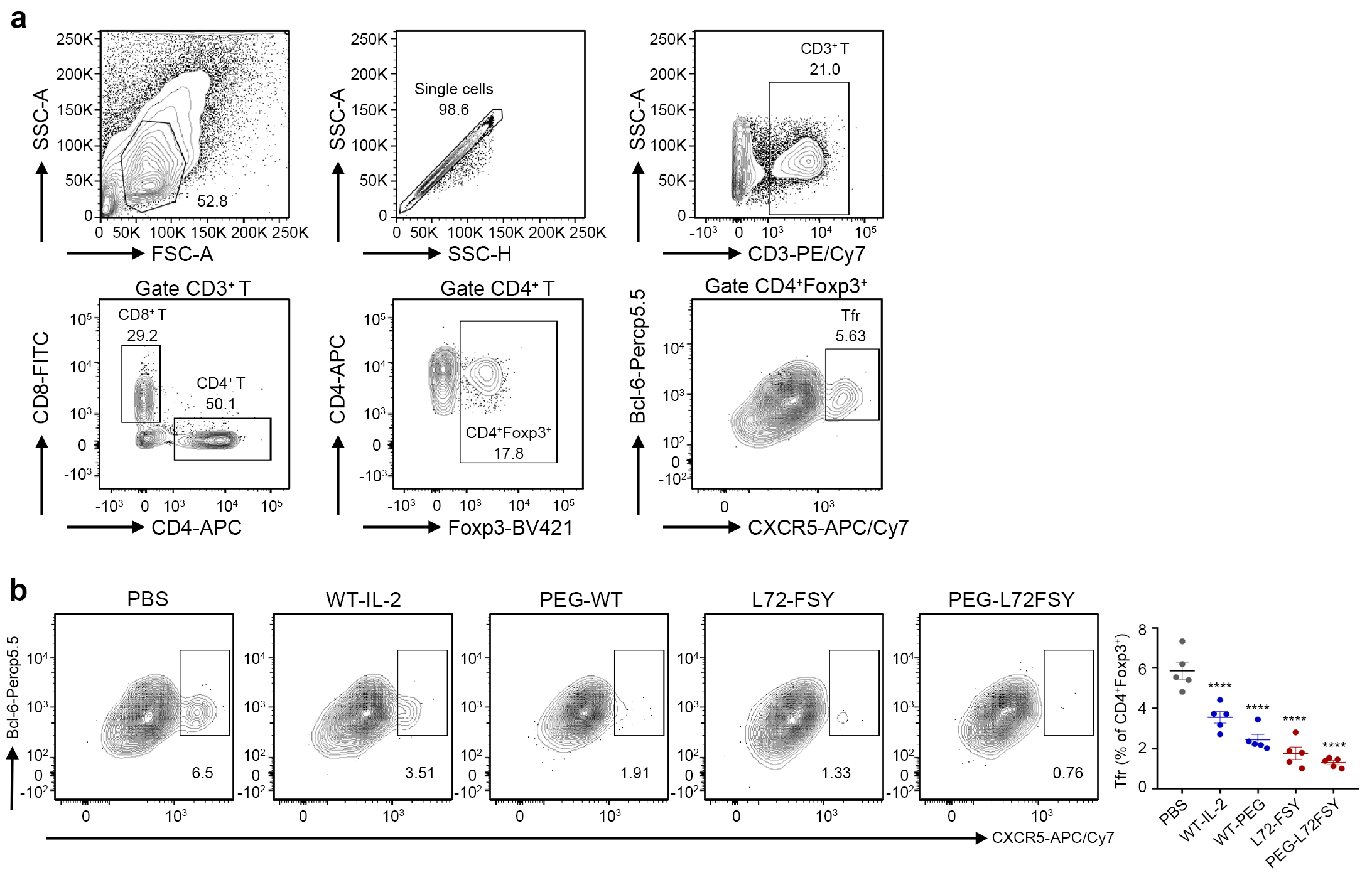


**Supplementary Figure 18** L72-FSY and PEG-L72-FSY treatment inhibit Tfr cell differentiation in pristane-treated B-hIL2RA mice. **a** Representative flow plots of the gating strategy. Tfr cells were defined as CD3^+^CD8^-^CD4^+^Foxp3^+^CXCR5^+^Bcl-6^+^ cells. **b** Representative flow plots and bar graphs reflecting the percentage change in Tfr cells among CD4^+^Foxp3^+^T cells after the indicated treatment. The data are presented as the mean ± SEM of 5 mice (n=5), and the p values were determined by one-way ANOVA (Dunnett’s multiple-comparison test compared with the PBS group). ****p ≤ 0.0001, See also Fig. 6b.

**Supplementary Table 1. Summary of in vitro bioactivities of WT-IL-2 versus L72-FSY**

| Variant | WT-IL-2 | L72-FSY |
| --- | --- | --- |
| Kon (1/Ms) | 3.89E+06 | 1.39E+06 |
| Kdis (1/s) | 1.30E-02 | 1.42E-02 |
| KD (nM) | 3.33 | 10.3 |
| EC50 (pg/ml) | 48.2 | 94.7 |

**Supplementary Table 2. Summary of antibodies used in CyTOF experiments**

| No. | Antibody | Metal channel | No. | Antibody | Metal channel |
| --- | --- | --- | --- | --- | --- |
| 1 | CD45 | 89Y | 20 | PD1 | 161Dy |
| 2 | CD44 | 113Ln | 21 | CXCR3 | 162Dy |
| 3 | CD3e | 115ln | 22 | CXCR5 | 163Dy |
| 4 | CD103 | 141Pr | 23 | CD86 | 164Dy |
| 5 | MHC_II | 142Nd | 24 | Ly6G | 165Ho |
| 6 | KLRG1 | 143Nd | 25 | Ki67 | 169Tm |
| 7 | CX3CR1 | 144Nd | 26 | Tbet | 170Er |
| 8 | CD24 | 145Nd | 27 | GATA3 | 171Yb |
| 9 | CD27 | 146Nd | 28 | CD127 | 172Yb |
| 10 | CD80 | 147Sm | 29 | Granzyme B | 173Yb |
| 11 | Ly6C | 148Nd | 30 | CTLA4 | 174Yb |
| 12 | hCD25 | 149Sm | 31 | CCR6 | 175Lu |
| 13 | CD49b | 150Nd | 32 | Siglec-H | 176Yb |
| 14 | CD62L | 151Eu | 33 | CD4 | 197Au |
| 15 | CD11c | 152Sm | 34 | CD8 | 198pt |
| 16 | IgD | 153Eu | 35 | CD11b | 209Bi |
| 17 | CD73 | 154Sm | 36 | NKp46 | 157Gd |
| 18 | CCR7 | 155Gd | 37 | F4/80 | 159Tb |
| 19 | BST2 | 156Gd | 38 | CD206 | 160Gd |

**Supplementary Table 3. Comparison of in vitro bioactivities of WT-IL-2 and PEG-L72FSY**

| Sample ID | IL-2Rα | | | IL-2Rβ | | | EC50 (ng/ml)^2^ | | Selectivity^3^ |
| --- | --- | --- | --- | --- | --- | --- | --- | --- | --- |
|  | Kon  (1/Ms) | Kdis  (1/s) | KD (nM) | Kon  (1/Ms) | Kdis  (1/s) | KD (nM) | Tregs | CD8+T |  |
| IL-2^1^ | 3.89E+06 | 1.30E-02 | 3.33 | 9.06E+05 | 7.47E-02 | 82.4 | 0.8±  0.27 | 2.7±  0.39 | 3.3 |
| L72-FSY | 1.39E+06 | 1.42E-02 | 10.3 | 2.15E+05 | 1.51E-02 | 70.0 | - | - | - |
| PEG-WT | 1.43E+06 | 4.51E-02 | 31.5 | 1.80E+05 | 1.46E-01 | 812 | - | - | - |
| PEG-L72FSY | 1.04E+06 | 4.70E-02 | 45.1 | 2.68E+05 | 1.99E-01 | 743 | 4.1±  1.47 | 28.1±  1.81 | 6.8 |

^1^ prepared by us; ^2^ Data are presented as mean ± SD; ^3^ defined as EC50 ration of CD8^+^ T to Treg cells

**Supplementary Table 4. Pharmacokinetic parameters of WT-IL-2 and PEG-L72FSY in two strains.**

| Strain | Variants | AUC^1^0-∞  (mg/L*h) | Tmax^2^  (h) | MRT^3^  (h) | T_1/2_^4^  (h) | Cl^5^  (mL/h) |
| --- | --- | --- | --- | --- | --- | --- |
| B-hIL2RA | WT-IL-2 | 0.03±0.003 | 0.5 | 2.2±0.29 | 0.6±0.21 | 172.5±17.42 |
|  | L72-FSY | 0.05±0.008 | 0.5 | 4.4±0.45 | 3.2±0.59 | 94.5±16.25 |
|  | PEG-WT | 0.4±0.03 | 4 | 31.1±1.41 | 19.4±0.88 | 11.7±1.40 |
|  | PEG-L72FSY | 0.4±0.04 | 4 | 39.2±3.68 | 26.0±3.55 | 12.7±1.55 |
| C57BL/6 | WT-IL-2 | 0.03±0.002 | 0.5 | 1.6±0.15 | 0.5±0.06 | 182.1±13.12 |
|  | L72-FSY | 0.03±0.006 | 0.5 | 1.5±0.22 | 0.6±0.13 | 200.2±41.74 |
|  | PEG-WT | 18.3±1.94 | 4 | 14.3±0.74 | 9.1±0.93 | 27.5±3.14 |
|  | PEG-L72FSY | 18.2±0.37 | 4 | 14.1±0.66 | 8.4±0.47 | 27.4±0.66 |

^1^ AUC: Area under drug concentration versus time curve; ^2^ Tmax：time of maximal drug concentration; ^3^ MRT: mean residence time; ^4^. T_1/2_: half-life of clearance; ^5^ Cl/f: apparent total plasma clearance. Data are presented as mean ± SD.
